# The profiling of extracellular vesicle subtypes in Huntington’s disease brains identifies Alix as a novel marker of neuropathology

**DOI:** 10.1186/s40478-025-02187-6

**Published:** 2025-12-08

**Authors:** Rocío Pérez-González, Anna Vázquez-Oliver, Nil Salvat-Rovira, Saül Martínez-Horta, Elisa Rivas-Asensio, Eva Borràs, Samanta Ortuño-Miquel, María Sánchez-Carcelén, Marta Garcia-Forn, Frederic Sampedro, Jesús Pérez-Pérez, Eduard Sabidó, Esther Pérez-Navarro, Jaime Kulisevsky

**Affiliations:** 1 Instituto de Investigación Sanitaria y Biomédica de Alicante (ISABIAL), Hospital Universitario Doctor Balmis de Alicante, Av. Pintor Baeza 12, 03010 Alicante, Spain; 2 Instituto de Neurociencias de Alicante, Universidad Miguel Hernández-CSIC, 03550 San Juan de Alicante, Spain; 3 Centro de Investigación en Red-Enfermedades Neurodegenerativas (CIBERNED), 28031 Madrid, Spain; 4 Movement Disorders Unit, Neurology Department, Hospital de la Santa Creu i Sant Pau, Institut de Recerca Sant Pau - Centre CERCA, 08041 Barcelona, Spain; 5 Departament de Medicina, Universitat Autònoma de Barcelona, 08193 Barcelona, Spain; 6 Center for Genomic Regulation (CRG), Barcelona Institute of Science and Technology, 08003 Barcelona, Spain; 7 Universitat Pompeu Fabra (UPF), 08003 Barcelona, Spain; 8 Departament de Biomedicina, Facultat de Medicina i Ciències de la Salut, Institut de Neurociències, Universitat de Barcelona, 08036 Barcelona, Spain; 9 Institut d’Investigacions Biomèdiques August Pi i Sunyer (IDIBAPS), 08036 Barcelona, Spain; 10 Neuroradiology Section, Radiology Department, Hospital Vall d’Hebron - Institut de Diagnostic Per la Imatge, 08035 Barcelona, Spain

**Keywords:** Huntington’s disease, Exosomes, Ectosomes, Alix, Annexin A2, Neurodegeneration

## Abstract

**Background:**

Huntington’s disease (HD) is the most frequent autosomal dominant neurodegenerative disorder, which is caused by a CAG repeat expansion in the HTT gene. Despite its well-defined genetic origin, there is currently no cure, and reliable biomarkers for disease progression and pathophysiology remain limited. Mutant huntingtin protein accumulates in endosomal compartments, disrupting endosomal trafficking and potentially affecting the biogenesis, release, and cargo of exosomes–extracellular vesicles (EVs) derived from the endosomal pathway. However, the role of exosomes in HD pathogenesis and their potential as biomarkers has been underexplored. In this work, we investigated whether the levels and content of small EV subpopulations, including exosomes, are altered in the brains of HD patients.

**Methods:**

We analyzed two distinct subpopulations of small EVs from the striatum and cortex of postmortem HD brains at early and advanced neuropathological stages, as well as from age-matched controls. EVs were isolated by differential ultracentrifugation and high-resolution iodixanol density gradient centrifugation, and analyzed by Western blotting, electron microscopy, NTA, and proteomics using mass spectrometry. EV secretion was also analyzed in primary fibroblasts derived from HD patients and healthy controls.

**Results:**

Mass spectrometry data revealed HD-associated alterations in EV protein content, particularly proteins related to the endosomal system. Our data also indicate that the level of ectosomes increased in the HD cortex, whereas exosomes were reduced in the HD striatum compared to controls. In terms of EV content, EVs from HD brains showed increased levels of Annexin A2 and decreased levels of Alix, a key component of the endosomal sorting complex required for transport (ESCRT). Alix depletion in EVs mirrored a progressive reduction of Alix in brain tissue, correlating with disease severity based on Vonsattel staging. In vitro, HD fibroblasts secreted EVs with reduced Alix content, despite no significant difference in cellular Alix levels compared to controls.

**Conclusions:**

These findings highlight disease-specific changes in EV populations and cargo in HD, and identify Alix as a potential neuropathological marker. This study advances our understanding of the role of brain-derived EVs in HD and underscores their potential utility in biomarker discovery.

**Supplementary Information:**

The online version contains supplementary material available at 10.1186/s40478-025-02187-6.

## Background

Huntington’s disease (HD) is an inherited, autosomal dominant neurodegenerative disorder characterized by progressive degeneration of the striatum and cerebral cortex [1]. The disease typically manifests in mid-adulthood with a triad of motor dysfunction, cognitive decline, and psychiatric disturbances, and progresses over 15–25 years [2]. The mutation responsible for HD is an abnormal expansion of a CAG repeat, coding for a polymorphic polyglutamine in the *HTT* gene that encodes for mutant huntingtin (HTT), a large protein of 3,144 amino acids [3]. Although the genetic cause of HD is well established, the disease exhibits marked clinical heterogeneity, and no disease-modifying therapies have been approved to date [4], though several therapeutic candidates are currently being evaluated in clinical trials [5]. Therefore, there is a pressing need for additional biomarkers that can both capture disease heterogeneity and serve as reliable tools for assessing therapeutic efficacy in clinical trials.

Although the specific mechanisms by which mutant HTT causes neuronal dysfunction and/or death remain unclear, compelling evidence suggests its involvement in altered endosomal trafficking in neurons [6–9]. Notably, such alterations are detectable in the pre-symptomatic stage of HD [10]. Impairment of the endosomal pathway can affect the production, secretion, and content of exosomes [11, 12], however, very limited research has been conducted to characterize exosomes in the disease.

Exosomes are a specific subtype of small extracellular vesicles (EVs) that are secreted into the extracellular space and biological fluids. They are enclosed by a phospholipid bilayer and carry proteins, lipids, and nucleic acids that reflect the physiological state of their cells of origin. Exosomes are formed within late endosomes or multivesicular bodies (MVBs) through the inward budding of the endosomal membrane, a process mediated by either endosomal sorting complex required for transport (ESCRT)-dependent or -independent mechanisms. The ESCRT accessory protein Alix (ALG-2 interacting protein), which links sorted cargo to ESCRT-III components, plays a key role in exosome biogenesis [13] and is commonly found in EVs [14]. Exosomes typically range from 50 to 200 nm in diameter, which may overlap in size with other EVs like ectosomes. Unlike exosomes, ectosomes are generated by direct budding from the plasma membrane and are enriched in Annexins, particularly Annexin A1 and Annexin A2. Notably, their density profiles differ from that of Alix [15], allowing for the distinction between these EV subtypes.

Given their distinct origins, exosomes and ectosomes may play different roles in maintaining brain homeostasis and differentially contribute to the progression of neurodegenerative diseases. Therefore, investigating EV heterogeneity, particularly exosomes that relate to alterations in the endosomal pathway, can provide valuable insights into the physiological and pathological processes underlying complex brain diseases like HD. Furthermore, due to the properties of brain-derived EVs as source of biomarkers [16], analyzing their cargo composition may not only reveal novel prognostic markers for HD but also serve as a tool for monitoring therapeutic responses.

In this study, we investigated whether the levels of two distinct small EV subtypes differ between HD and healthy individuals by analyzing their abundance and protein composition in post-mortem brains at two stages of HD-related neurodegeneration. This approach enabled the identification of key alterations in the EV physiopathology in HD, and identified Alix as a novel marker of neuropathology.

## Methods

### Human post-mortem tissues

Frozen post-mortem striatum (putamen and caudate nucleus) and dorsolateral frontal cortex (hereafter cortex) tissues and data from HD patients and non-mutation carriers were provided by the HCB-IDIBAPS Biobank (B.0000575), integrated in the Platform ISCIII Biobanks and Biomodels and they were processed following standard operating procedures with the appropriate approval of the Ethics and Scientific Committees. The HD brains were neuropathologically assessed by the Vonsattel grading system based on the macroscopic appearance of striatal atrophy and, at a microscopic level, on the striatal neuronal loss and degree of reactive astrogliosis [17, 18]. Details of the brain tissues used for this study are described in Table 1.

**Table 1:** Huntington’s disease and control post-mortem brain tissues used in this study

Lab ID	Experimental group	Vonsattel grade	CERAD	BRAAK stage	Age	Sex	PMD (hh:mm)	Striatum EVs	Cortex EVs
1	Control	0	None	3	86	M	7:25	Y	Y
2	Control	0	NA	1	76	M	6		Y
3	HD(1 + 2)	1	NA	NA	73	M	7	Y	Y
4	HD(1 + 2)	2–3	moderate	4	68	M	6:10	Y	Y
5	HD(3 + 4)	3–4	NA	NA	55	M	7	Y	Y
6	HD(3 + 4)	3	NA	NA	56	M	4:30		Y
7	Control	0	NA	2	78	M	7:30	Y	Y
8	Control	0	B	2	86	M	7:25	Y	Y
9	HD(1 + 2)	2	moderate	6	76	M	6	Y	Y
10	HD(1 + 2)	2	moderate	5	72	M	13:10		Y
11	HD(3 + 4)	2–3	sparse	1	84	M	8	Y*	Y
12	HD(3 + 4)	3	none	2	69	M	12:30		Y
13	Control	0	NA	NA	86	F	4	Y	Y
14	Control	0	none	1–2	56	F	14		Y
15	HD(1 + 2)	2	NA	NA	69	F	15:30	Y	Y
16	HD(1 + 2)	2	moderate	3	86	F	12:20		Y
17	HD(3 + 4)	3	none	1	65	F	6:30	Y	Y
18	HD(3 + 4)	3	NA	2	69	F	12:30	Y*	Y
19	Control	0	B	5	77	F	8:26		
20	Control	0	B	5	78	M	18:05		
21	Control	0	1	2	86	F	17:15		
22	Control	0	NA	1	62	M	4:30		
23	Control	0	3	NA	75	F	5:00		
24	HD(3 + 4)	2–3	NA	1	54	F	7:30		
25	HD(1 + 2)	2	sparse	1	59	F	4:55		
26	HD(1 + 2)	2	0	1	62	M	14:30		
27	HD(1 + 2)	2	absent	1	66	M	16:42		
28	HD(1 + 2)	2–3	sparse	1	65	F	6:15		
29	HD(3 + 4)	3	NA	NA	33	M	6:08		

NA, Not available; M, Male; F, Female. Y, Yes, tissue availability for EV work

*Striatum samples 11 and 18 were pooled to reach the minimum amount of tissue required for processing

### Isolation of small EV subpopulations from brain tissues

Small EVs were isolated from an average of 340 mg of striatum or 440 mg of cortex from HD patients at early (Vonsattel grades 1–2) or late (Vonsattel grades 3–4) neuropathological stages, as well as from healthy non-mutation carriers. Isolation was performed as previously described [19] using a high-resolution iodixanol gradient [20, 21]. Briefly, the tissues were minced and incubated with 20 U/mL papain (Worthington Biochemical Corporation) in Hibernate A medium (ThermoFisher Scientific) for 15 min at 37 °C. The enzymatic digestion was stopped by the addition of ice-cold Hibernate A supplemented with a cocktail of protease and phosphatase inhibitors (Halt™ Protease and Phosphatase Inhibitor Cocktail, ThermoFisher Scientific). The solution was gently dissociated by pipetting and centrifuged at 300×*g* for 10 min at 4 °C to pellet undigested tissue and intact cells. The supernatant was subsequently filtered through a 40 μm cell strainer and then centrifuged at 2,000×*g* for 10 min at 4 °C to discard large debris and apoptotic bodies. The resulting supernatant was centrifuged at 10,000×*g* for 30 min at 4 °C to discard smaller debris, passed through a 0.2 μm surfactant-free cellulose acetate (SFCA) membrane filter and ultra-centrifuged at 100,000×*g* for 70 min at 4 °C to pellet the crude EVs in a Kontron TFT 55.38 fixed-angle rotor (k factor 51). The pellet was washed in phosphate-buffered saline (PBS) pH 7.4, re-centrifuged at 100,000×*g* for 70 min at 4 °C and resuspended in a 40% v/v OptiPrep (iodixanol) solution, containing 10 mM Tris–HCl pH7.4, 0.25 M sucrose and 40% iodixanol (all reagents from Sigma-Aldrich). An OptiPrep density step-gradient was set up by carefully layering on the top of the 40% OptiPrep-equilibrated EVs a decreasing scale of OptiPrep solutions (20%, 15%, 13%, 11%, 9%, 7%, 5%). The column was centrifuged overnight at 4 °C in a swinging bucket SW41Ti rotor at 200,000×*g*. Afterwards, 1.5 ml fractions (8 fractions in total), corresponding to the different interphases, were collected, washed in PBS and centrifuged at 100,000×*g* for 70 min at 4 °C in a Kontron TFT 55.38 fixed-angle rotor. Pellets were resuspended in 30 µl of PBS and analyzed as indicated below. The low-density fractions 1 to 3 (Fr1-3) and the intermediate-density fractions 4 to 7 (Fr4-7) were pooled for downstream analyses.

### Nanoparticle tracking analyses

The particle size distribution and concentration measurements were evaluated with a Nanosight NS300 (Particle Tracking Analysis) instrument (Malvern Panalytical, Malvern, UK). The vesicles were resuspended in PBS and diluted to the working range of the system (10^6^–10^9^ particles/ml). Videos were captured and analysed with the Nanosight NS300 software (version 3.4) using a sCMOS camera.

### Cryogenic electron microscopy

A volume of 3.9 µl of EV suspension was blotted onto 400 mesh lacey carbon grids (© Micro to Nano 2021) previously glow discharged in a PELCO easiGlow discharge unit. Next, the sample was plunged into liquid ethane (− 175 °C) by means of a Leica EM GP cryo-work station and subsequently transferred and visualized in a Jeol JEM-2011 TEM electron microscope operating at 200kV. Samples were maintained at − 175 °C during their observation and captures were obtained with a Gatan CCD 895 ultrascan camera. Image processing was performed using ImageJ software.

### LC–MS/MS analysis

#### Sample preparation

EVs were lysed in 6 M Urea in the bioruptor for 15 min. Samples were reduced with dithiothreitol (30 nmol, 37 °C, 60 min) and alkylated in the dark with iodoacetamide (60 nmol, 25 °C, 30 min). The resulting protein extract was first diluted to 2 M urea with 200 mM ammonium bicarbonate for digestion with endoproteinase LysC (1:10 w:w, 37 °C, 6 h), and then diluted twofold with 200 mM ammonium bicarbonate for trypsin digestion (1:10 w:w, 37 °C, o/n). After digestion, peptide mix was acidified with formic acid and desalted with a MicroSpin C18 column (The Nest Group, Inc) prior to LC–MS/MS analysis.

#### Chromatographic and mass spectrometric analysis

Samples were analyzed by LC–MS/MS in an Orbitrap Eclipse mass spectrometer coupled to an EASY-nLC 1200 (Thermo Fisher Scientific). Peptides were loaded directly onto the analytical column and were separated by reversed-phase chromatography using a 50-cm column with an inner diameter of 75 μm, packed with 2 μm C18 particles. Chromatographic gradients started at 95% buffer A and 5% buffer B with a flow rate of 300 nl/min and gradually increased to 25% buffer B and 75% A in 79 min and then to 40% buffer B and 60% A in 11 min. After each analysis, the column was washed for 10 min with 100% buffer B. Buffer A: 0.1% formic acid in water. Buffer B: 0.1% formic acid in 80% acetonitrile. The mass spectrometer was operated in positive ionization mode with nanospray voltage set at 2.4 kV and source temperature at 305 °C. The acquisition was performed in data-dependent acquisition (DDA) mode and full MS scans with 1 micro scans at resolution of 120,000 were used over a mass range of m/z 350–1400 with detection in the Orbitrap mass analyzer. Auto gain control (AGC) was set to ‘standard’ and injection time to ‘auto’. In each cycle of data-dependent acquisition analysis, following each survey scan, the most intense ions above a threshold ion count of 10,000 were selected for fragmentation. The number of selected precursor ions for fragmentation was determined by the “Top Speed” acquisition algorithm and a dynamic exclusion of 60 s. Fragment ion spectra were produced via high-energy collision dissociation (HCD) at normalized collision energy of 28% and they were acquired in the ion trap mass analyzer. AGC was set to 2E4, and an isolation window of 0.7 m/z and a maximum injection time of 12 ms were used. Digested bovine serum albumin was analyzed between each sample to avoid sample carryover and to assure stability of the instrument and QCloud [22] has been used to control instrument longitudinal performance during the project.

#### Data analysis

Acquired spectra were analyzed using the Proteome Discoverer software suite (v2.4, Thermo Fisher Scientific) and the Mascot search engine (v2.6, Matrix Science [23]). The data were searched against a Swiss-Prot human database (as in June 2020) plus a list [24] of common contaminants and all the corresponding decoy entries. For peptide identification a precursor ion mass tolerance of 7 ppm was used for MS1 level, trypsin was chosen as enzyme, and up to three missed cleavages were allowed. The fragment ion mass tolerance was set to 0.5 Da for MS2 spectra. Oxidation of methionine and N-terminal protein acetylation were used as variable modifications whereas carbamidomethylation on cysteines was set as a fixed modification. False discovery rate (FDR) in peptide identification was set to a maximum of 5%. Protein abundances were estimated using the three most intense peptides per protein and normalized based on total peptide amount. Venn diagram was performed with Venny 2.0. The identification of Gene Ontology categories by cellular component or biological process [25] pathways were performed with ShinyGO 0.82 [26], and the network of proteins by STRING 12.0. The raw proteomics data have been deposited to the PRIDE repository [27] with the project accession number PXD060574.

### Fibroblasts

Foreskin untransformed primary fibroblasts from HD patients and healthy subjects were either derived from sterile, non-necrotic skin biopsies and processed as previously described [28] or purchased from the Coriell Institute (catalogue numbers: GM04723, GM04847, GM04837, GM03440 and GM08399). Fibroblast cell details are described in Table 2. The cells were grown at 37 °C in Dulbecco’s modified Eagle’s medium (DMEM), supplemented with 10% (v/v) fetal bovine serum, penicillin (100 U/ml) + streptomycin (100 µg/ml), and 2 mM GlutaMAX (all reagents from Thermo Fisher Scientific) at 5% CO_2_ in a humidified incubator. EV-depleted cell medium was obtained by ultracentrifugation of supplemented media at 100,000×*g* for 16 h at 4 °C in a Kontron TFT 55.38 fixed-angle rotor followed by filtration of the supernatant through a 0.2 µm pore size filter for sterilization. All experiments were performed using cells at passage 13 or lower (starting from frozen stocks at passage 8).

**Table 2:** Fibroblasts used in this study

Source	Genotype	Sex	Years at sampling	Years at onset of symptoms
Coriell Institute	Control	Male	20	
Coriell Institute	Control	Female	19	
Skin biopsy	Control	Female	53	
Coriell Institute	HD	Female	19	14
Coriell Institute	HD	Male	31	37
Coriell Institute	HD	Male	23	30
Skin biopsy	HD	Male	43	38

### Isolation of EVs from fibroblast conditioned media

Fibroblasts at 80% confluence in 150 mm plates (Corning Incorporated) were grown in EV-depleted medium for 24 h. The medium was collected and centrifuged at 300×*g* for 10 min at 4 °C to discard cells or cell fragments. The supernatant was serially centrifuged at 2,000×*g* for 10 min and at 10,000×*g* for 30 min at 4 °C to discard cell debris. The supernatant was then ultra-centrifuged at 100,000×*g* for 70 min at 4 °C in a Kontron TFT 55.38 fixed-angled rotor and the pellet resuspended in PBS and re-centrifuged at 100,000×*g* for 70 min at 4 °C. The EV pellet was collected in PBS and prepared for Western blot analysis as described below.

### Sample homogenization, protein estimation and Western blot analyses

EVs in PBS were lysed in 2X RIPA buffer (50 mM Tris HCl-pH 7.4, 1% Triton-X, 1% Sodium deoxycholate, 0.1% SDS, 150 mM NaCl, 1 mM EDTA and a cocktail of protease and phosphatase inhibitors (ThermoFisher Scientific)), sonicated for 45 s and kept on ice for 20 min with vigorous vortex every 3–4 min. Approximately 50 mg of striatum or cortex were homogenized in ice-cold 1X RIPA with a disposable homogenising pestle, sonicated for 45 s, kept on ice for 30 min with vigorous vortex every 3–4 min, and centrifuged at 13,000×*g* for 15 min at 4 °C to clear the homogenates. Fibroblast lysates were prepared by scrapping the cells in 1X RIPA. Total protein levels of EV, brain or fibroblast lysates were estimated by using the Pierce BCA (bicinchoninic acid) Protein Assay Kit (ThermoFisher Scientific) according to the manufacturer’s protocol, and the colorimetric reaction was quantified as the absorbance at 570 nm using the AD 340C microplate reader (Beckman Coulter). The samples examined by Western blot were supplemented with a 4X Laemmli sample buffer (Bio-Rad), boiled for 10 min at 90 °C and loaded into either 10% or gradient 4–20% stain-free acrylamide gels (Bio-Rad) to run at 80 V for 30 min followed by 200 V for 40 min. After the electrophoresis, the gels were imaged for total protein measurement in a ChemiDoc XRS system (Bio-Rad). The proteins were transferred onto PVDF membranes (Bio-Rad) through the semi-dry system (trans-blot turbo, Bio-Rad), blocked in Everyblot blocking buffer (Bio-Rad) and incubated overnight with antibodies to Alix (1:1000, Millipore), Annexin A2 (1:1000, Abcam), TSG101 (1:1000, Thermofisher Scientific), CD9 (1:1000, Cell Signaling), CD81 (1:1000, Cell Signaling), Calnexin (Genetex; 1:1000) or Vinculin (Genetex; 1:1000). Membranes were washed in Tris-buffered saline with 0.1% Tween 20 (TBST) three times for 10 min and incubate for 1 h at room temperature with an anti-HRP secondary antibody diluted at 1:7500 in blocking buffer. After washing the membranes in TBST four times for 10 min, they were incubated with Femto ECL (Thermofisher Scientific) for 5 min. The protein bands were visualized through the GeneGnome XRQ chemiluminescence imaging system (Syngene) and quantified using ImageJ (NIH). 

### Statistical analysis

All analyses were performed on GraphPad Prism versions 8 or 9, except for partial correlations (corrected) by age, which were performed on SPSS Statistics 26.

## Results

### Separation of two distinct EV subtypes in human post-mortem brains

**Fig. 1:**
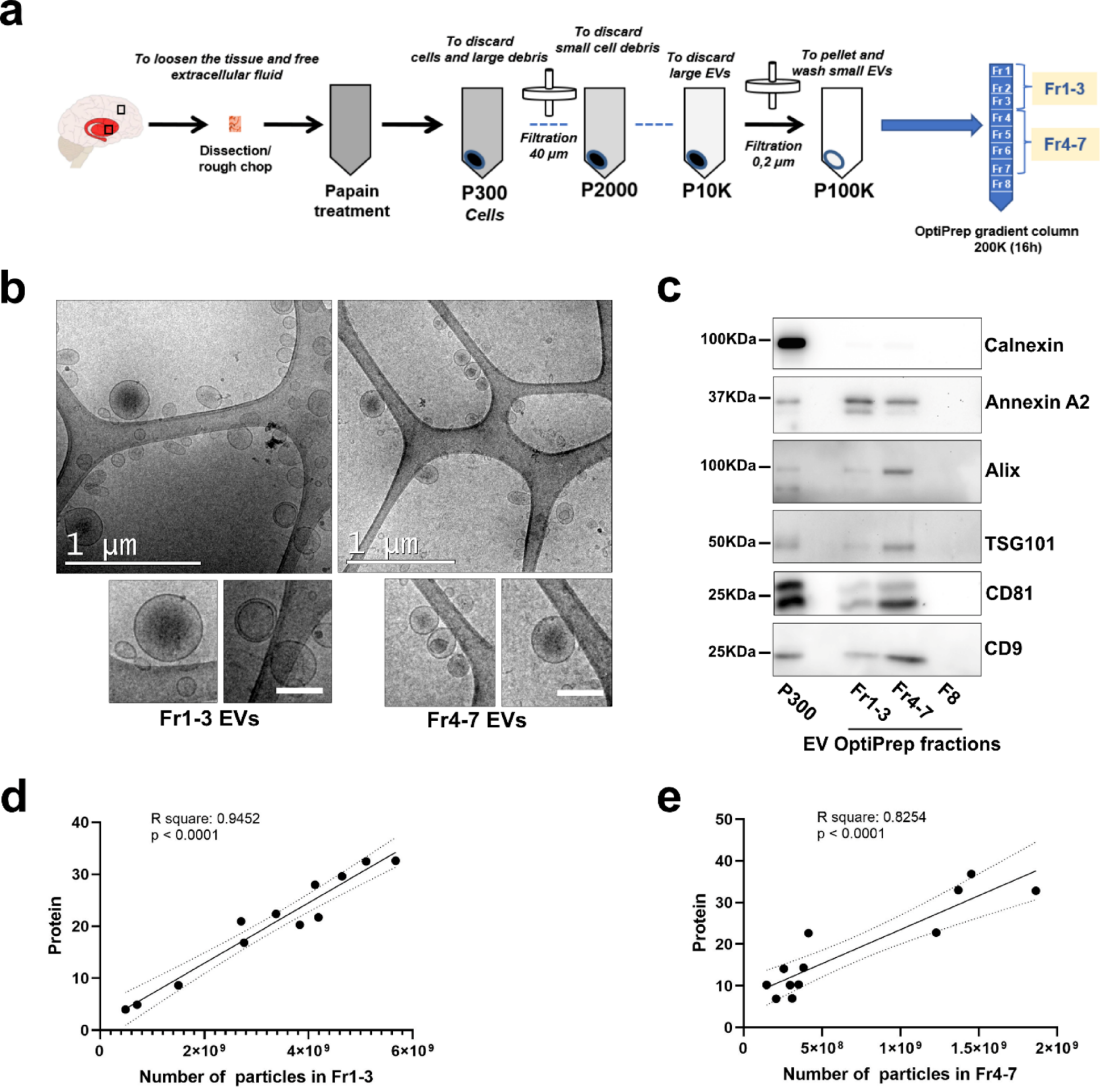
Purity of brain EV subtypes demonstrated by electron microscopy and protein-based analysis methods. **a** Flow-chart of the method used for EV isolation showing the different pellets obtained along the process (P300, after the 300×*g* spin; P2000, after the 2,000×*g* spin; P10K, after the 10,000×*g* spin; and P100K, after 100,000×*g* spin) and the gradient column. **b** Wide-field cryo-EM images with magnifications of striatum EVs underneath (scale bar: 100 nm) from Fr1-3 (left) and Fr4-7 (right) pooled fractions. **c** Representative Western blot showing the enrichment of the EV marker Annexin A2 in Fr1-3, and Alix, TSG101, CD9 and CD81 in Fr4-7. The absence of the intracellular marker Calnexin is also shown. P300 was used as positive control. The particle to protein ratio was used to estimate the purity of EVs in the pooled fractions Fr1-3 (**d**) and Fr4-7 (**e**). Both, the number of particles and protein levels were normalized to brain weight. R-squared and *P*-values are shown. Dashed-line indicate the 95% confidence bands of the best-fit line

Given the challenges associated when working with human post-mortem tissues, we first assessed the efficiency of our method for isolating EV subtypes from specific brain regions (striatum and cortex) of HD brains at both early and advanced stages of degeneration, as well as from control brains in our experimental sample set (Table 1). The isolation was performed using differential ultracentrifugation followed by purification with a high-resolution iodixanol density gradient (Fig. 1a). Throughout the isolation process, all intermediate pellets were collected and analyzed by Western blot to assess the purity of the 100.000 g pellet corresponding to crude or pre-gradient EVs. Crude EVs were enriched in Alix and Annexin A2 (Supplementary Fig. 1a–c), whereas Flotillin-1 was more abundant in the 10,000 g pellet (Supplementary Fig. 1a, d). CD9 and CD81, commonly used as EV markers but highly expressed in the plasma membrane [29], were enriched in the 300 g pellets corresponding to the cells (Supplementary Fig. 1a, e, f). In addition, Calnexin, an intracellular marker, was mostly excluded from EVs, confirming minimal intracellular contamination (Supplementary Fig. 1a, g). Following the purification of crude EVs in an iodixanol column, the lighter (1–3, Fr1-3) and intermediate fractions (4–7, Fr4-7) were used for downstream analysis. Cryogenic electron microscopy confirmed the purity of both pools of fractions (Fig. 1b), and western blot analysis revealed that Fr4-7 EVs were enriched in Alix, TSG101, CD9 and CD81, whereas Fr1-3 EVs contained higher levels of Annexin A2 (Fig. 1c). We included CD9 and CD81 in the panel of markers because tetraspanin expression can vary across different EV subtypes [30]. Additionally, protein to particle ratio was used to estimate the purity of the EVs [31–33]. EVs contained in Fr1-3 (Fig. 1d) and in Fr4-7 (Fig. 1e) showed strong correlations between protein amounts and particle numbers indicating the purity of the EV subtype preparations. Thus, by using this method, we achieved the separation of two distinct EV subtypes in accordance with the Minimal Information for Studies of Extracellular Vesicles (MISEV) guidelines [14, 34]. Although the specific origin of these subtypes is not demonstrated in this study, for simplicity, the pool of fractions Fr1-3 EVs will be referred to as ‘ectosomes’ due to their enrichment in Annexin A2, while Fr4-7 EVs will be called ‘exosomes’ based on their enrichment in Alix and TSG101.

### Proteomic profiling of cortical exosomes in HD

**Fig. 2:**
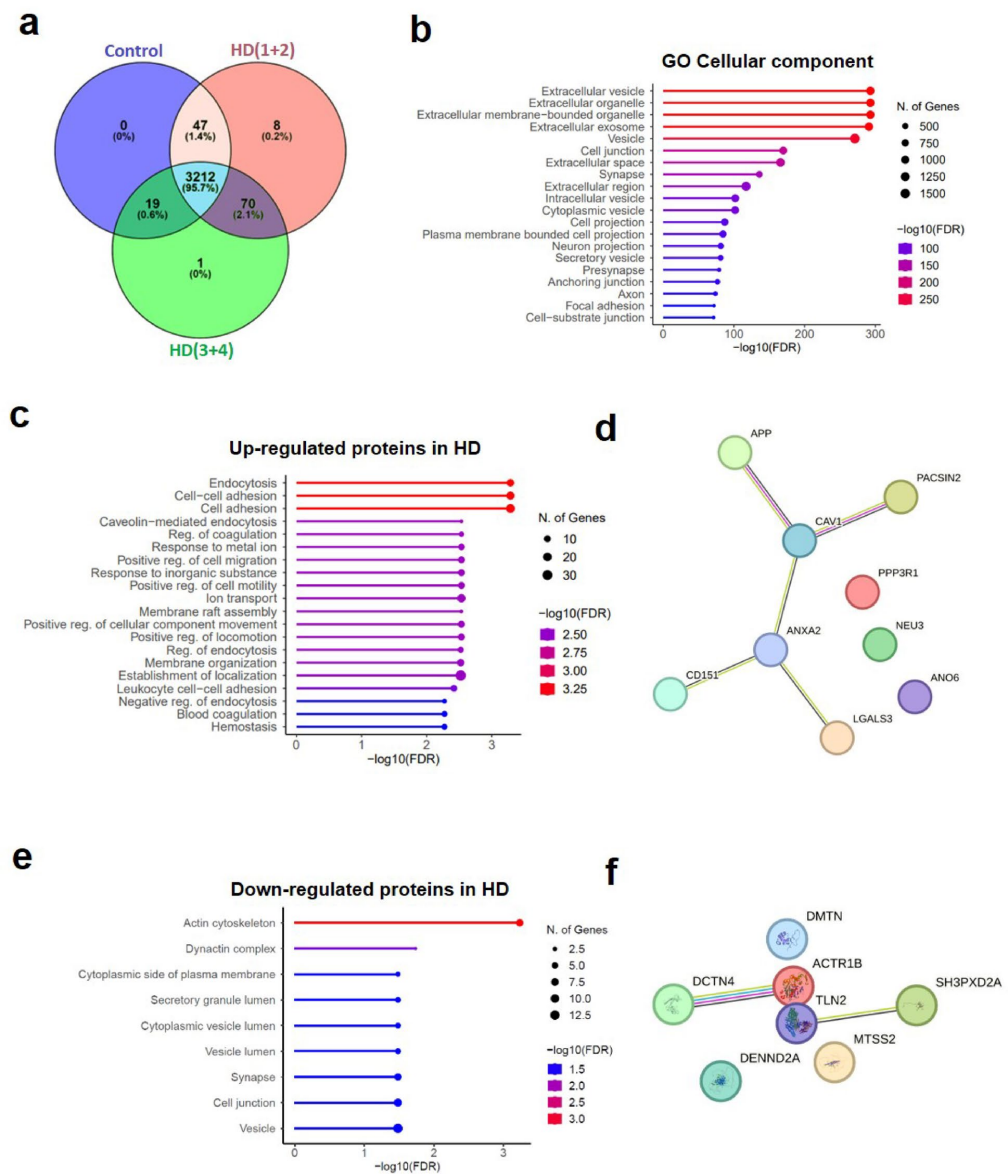
Proteomic analysis of cortical exosomes. **a** Venn diagram representing the number of EV proteins differentially identified in Controls, HD(1 + 2) and HD(3 + 4). **b** Gene ontology (GO) analysis shows the top cellular compartments represented by the common protein IDs identified in all groups. **c** GO analysis by biological process of a subset of up-regulated proteins in HD and **d** the network of proteins related to endocytosis. **e** GO analysis by biological process of down-regulated proteins in HD, and **f** network of proteins related to the top category (actin cytoskeleton). *n* = 6 Controls, *n* = 6 HD (1 + 2), *n* = 6 HD (3 + 4)

In order to specifically assess whether endosomal alterations had an impact on exosomes and further explore their protein content, the pool of fractions enriched in exosomes isolated from the HD cortex and controls were analyzed by mass spectrometry-based proteomics. We identified 3278 proteins in the control samples, 3337 proteins in HD(1 + 2) samples, and 3302 proteins in the HD(3 + 4) samples. Six biological replicates were analyzed per condition. Among them, 3212 proteins were detected in at least one biological replicate (Fig. 2a). The common proteins among the different groups were tested for properties pertaining to the “cellular component” and “biological process” ontology by Gene Ontology analysis. Regarding the cellular component, most of the proteins were found to be included in the “Extracellular vesicle”, “Extracellular organelle”, “Extracellular membrane-bounded organelle”, “Extracellular exosome” and “Vesicle” categories (Fig. 2b). Next, we conducted an enrichment analysis for proteins that met the significance threshold of *p* < 0.01 with a fold change > 2,8 among the experimental groups to capture cumulative effects of different proteins. Following this approach, 133 proteins were selected (Supplementary Table S1). Gene ontology analysis from the subset of 99 proteins that were up-regulated in HD vs Controls, or HD(3 + 4) vs HD(1 + 2) revealed that the top category was related to endocytosis (Fig. 2c), with Annexin A2 as one of the key proteins involved (Fig. 2d). On the other hand, 30 down-regulated proteins were mostly related to actin cytoskeleton (Fig. 2e, f). These data show that the EV proteome is altered in HD, reflecting changes particularly affecting endosomal vesicular transport and actin cytoskeleton remodelling pathways.

### EV subtypes alterations in the HD brain

**Fig. 3:**
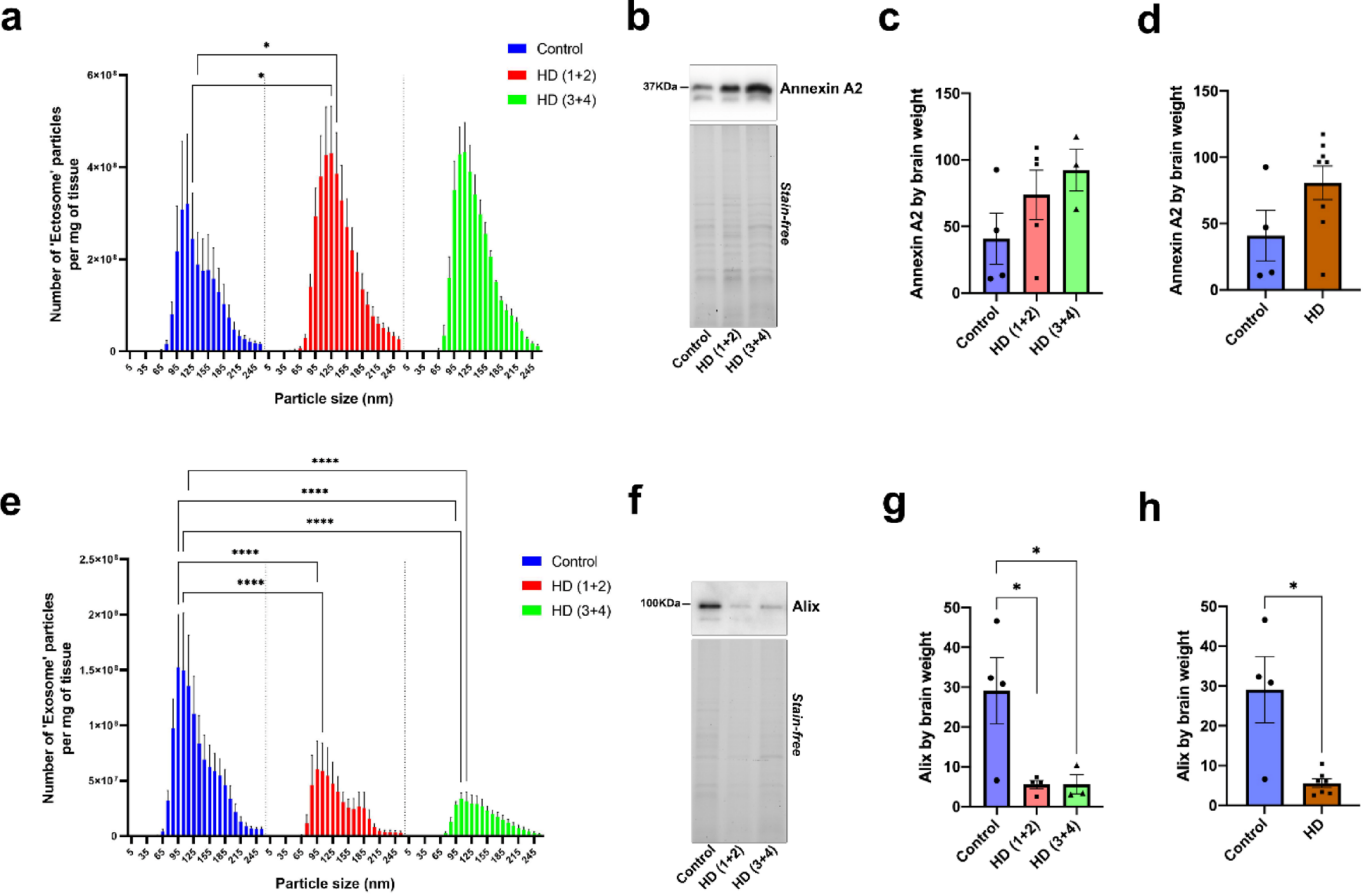
EV subtype profiling in HD striatum. **a** Number and size profiling of striatum Fr1-3 EVs estimated by NTA and normalized by brain weight. No significant interaction between the effect of particle size and the experimental group was found (F(198, 900) = 0.6376, *P* > 0.9999). Simple main effects analysis showed that the experimental group had a statistically significant effect on the number of particles (*P* = 0.0006). **b** Representative Western blot showing the specific band for Annexin A2. **c** Quantification of Annexin A2 in the three experimental groups. No significant main effect of the experimental group on Annexin A2 levels was detected (*P* = 0.2332). **d** Quantification of Annexin A2 in HD versus controls (*P* = 0,0727). **e** Number and size profiling of exosomes estimated by NTA and normalized by brain weight. A significant interaction between the effect of particle size and the experimental group was found (F(198, 900) = 2.458, *P* < 0.0001). Simple main effects analysis showed that both the experimental group and the particle size had an effect on the number of particles (*P* < 0.0001). Only differences between groups with *P* < 0.0001 are depicted. **f** Representative Western showing the specific band for Alix. **g** Quantification of Alix in the three experimental groups. A significant main effect of the experimental group on Alix levels was detected (*P* = 0.0217). **h** Quantification of Alix in HD versus controls (*P* < 0.05). In the Western blots the same volume of EV lysate per sample was loaded and the intensity of the bands was normalized by brain weight. Two-way ANOVA and ordinary one-way ANOVA followed by Tukey’s multiple comparisons test were used to analyze the NTA and the Western blot data, respectively, from the three experimental groups. When HD cases were combined Mann–Whitney tests were performed. *n* = 4 Controls, *n* = 5 HD (1 + 2), *n* = 3 HD (3 + 4), except for Alix analysis: *n* = 4 Controls, *n* = 4 HD (1 + 2), *n* = 3 HD (3 + 4) in which Grubb’s test identified one outlier in the HD(1 + 2) group. Data is shown as mean ± SEM (standard error of the mean). *P < 0.05, ****P < 0.0001

Since the proteomic analysis pointed to key alterations in HD EVs, we aimed to further investigate the levels of both, exosomes and ectosomes, in two key brain regions affected in HD, striatum and cortex, considering their distinct biogenesis pathways and molecular composition. To do so, we used nanoparticle tracking analysis (NTA), protein measurements and Western blot. When we compared the total number of particles (Supplementary Fig. 2) and the total amount of protein (Supplementary Fig. 3) in both EV subtypes, no significant differences were found among groups neither in the striatum nor in the cortex. Similar trends were observed when the data from all HD cases were combined for the number of particles (Supplementary Fig. 4) and amount of protein (Supplementary Fig. 5). However, when accounting for particle size, we found that the number of 125 and 135 nm particles in striatum ectosomes was significantly higher in HD(1 + 2) than in control brains (Fig. 3a). This increase in the number of particles might be partially explained by a trend in the increase of Annexin A2 in HD ectosomes compared to controls (Fig. 3b, c).

A similar trend was found when all HD cases were combined (Fig. 3d). When we analysed the levels of exosomes, we found a significant decrease of particles within the range of sizes from 85 to 125 nm in HD striatum, regardless the Vonsattel stage, compared to controls (Fig. 3e), with the more robust changes within particles of 95, 105, and 115 nm. This decrease in particles was most likely due to the significant reduction of the number of Alix-positive EVs in HD compared to controls (Fig. 3f–h).

**Fig. 4:**
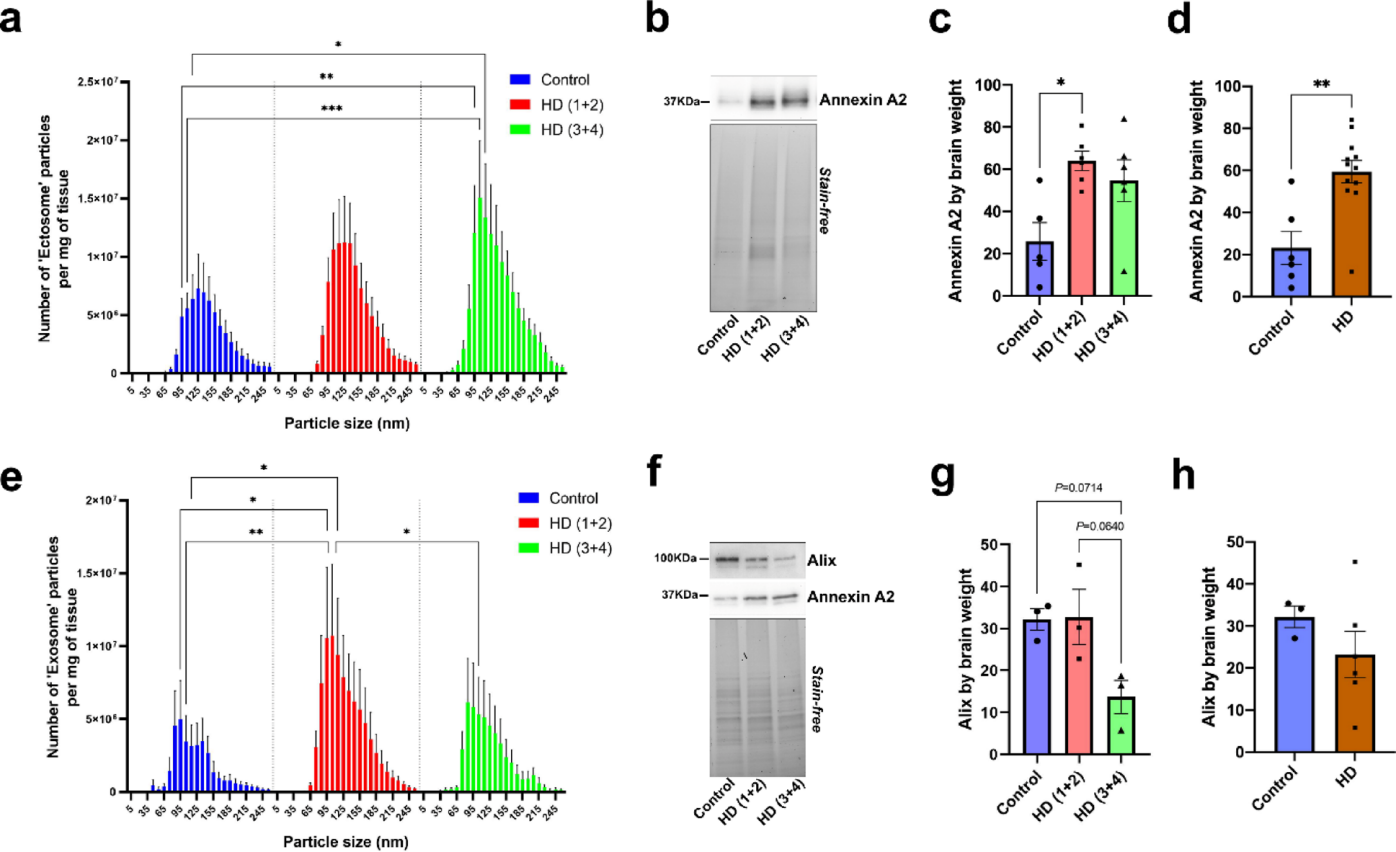
EV subtype profiling in HD dorsolateral frontal cortex. **a** Number and size profiling of cortical ectosomes estimated by NTA and normalized by brain weight. No significant interaction between the effect of particle size and the experimental group was found (*P* = 0.8144). Simple main effects analysis showed that both the experimental group and the particle size have a statistically significant effect on the number of particles (*P* < 0.0001). **b** Representative Western blot showing the specific band for Annexin A2. **c** Quantification of Annexin A2 in the three experimental groups. A significant main effect of experimental group on Annexin A2 levels was detected (*P* = 0.0147). **d** Quantification of Annexin A2 in HD versus controls (*P* = 0.0047). **e** Number and size profiling of cortical exosomes estimated by NTA and normalized by brain weight. No significant interaction between the effect of particle size and the experimental group was found (*P* = 0.8709). Simple main effects analysis showed that both the experimental group and the particle size have a statistically significant effect on the number of particles (*P* < 0.0001). **f** Representative Western blot showing the levels of Alix and Annexin A2. **g** Quantification of Alix in the three experimental groups. A significant main effect of the experimental group was detected on Alix levels (*P* = 0.0467) but not on Annexin A2 levels (*P* = 0.1194). **h** Quantification of Alix HD versus controls (*P* = 0.2619). In the Western blots, the same volume of EV lysate per sample was loaded and the intensity of the bands was normalized by brain weight. Two-way ANOVA and ordinary one-way ANOVA followed by Tukey’s multiple comparisons test were used to analyze the NTA and the Western blot data, respectively, from the three experimental groups. When HD cases were combined Mann–Whitney tests were performed.* n* = 5 controls, *n* = 6 HD (1 + 2), *n* = 6 HD (3 + 4) for Fr1-3 and *n* = 3 per group for Fr4-7. Data is shown as mean ± SEM. *P < 0.05, **P < 0.01, ***P < 0.001

In the case of the cortex, NTA revealed that the particles with a size between 95 and 115 nm were significantly increased in HD(3 + 4) compared to controls (Fig. 4a). This increase was consistent with a higher amount of Annexin A2-positive EVs that was only significant in the case of HD(1 + 2) (Fig. 4b, c). However, the level of Annexin A2 was clearly increased in HD relative to controls when combining all HD cases (Fig. 4d). Regarding cortical exosomes, a higher number of particles was detected in the range of sizes between 95 and 115 nm in HD(1 + 2) compared to controls (Fig. 4e). There was also a significant decrease of 95 nm particles in HD(3 + 4) compared to HD(1 + 2) (Fig. 4e). Given these differences in the particle count, we next assessed whether the level of Alix was also altered in HD. Western blot analysis revealed a trend to lower levels of Alix-positive EVs in HD(3 + 4) compared to controls (Fig. 4f, g). No significant changes were found either when analyzing all HD cases combined (Fig. 4h). The increase in the number of particles detected by NTA in cortex EVs was most likely due to the increase of Annexin A2-positive EVs that were also present in the exosome population (Fig. 4f).

To explore potential mechanisms underlying the altered release of EVs in HD brains, we examined whether changes in Annexin A2 and Alix protein levels were associated with variations in vesicle counts. Correlation analyses revealed strong positive associations between Annexin A2 (Supplementary Fig. 6a) and Alix (Supplementary Fig. 6b) and the number of exosome particles in the striatum, suggesting that Annexin A2 and Alix dysregulation may directly contribute to altered EV biogenesis or release in this region. On the contrary, no significant correlations were found in the cortex, either for Annexin A2 (Supplementary Fig. 6c) or Alix (Supplementary Fig. 6d).

Taken together, these data indicate that EV changes in HD are most likely brain region- and EV subtype- dependent: while the level of ectosomes is increased in the HD cortex, the level of exosomes is reduced in the striatum.

### Altered content of Annexin A2 and Alix in HD EVs

**Fig. 5:**
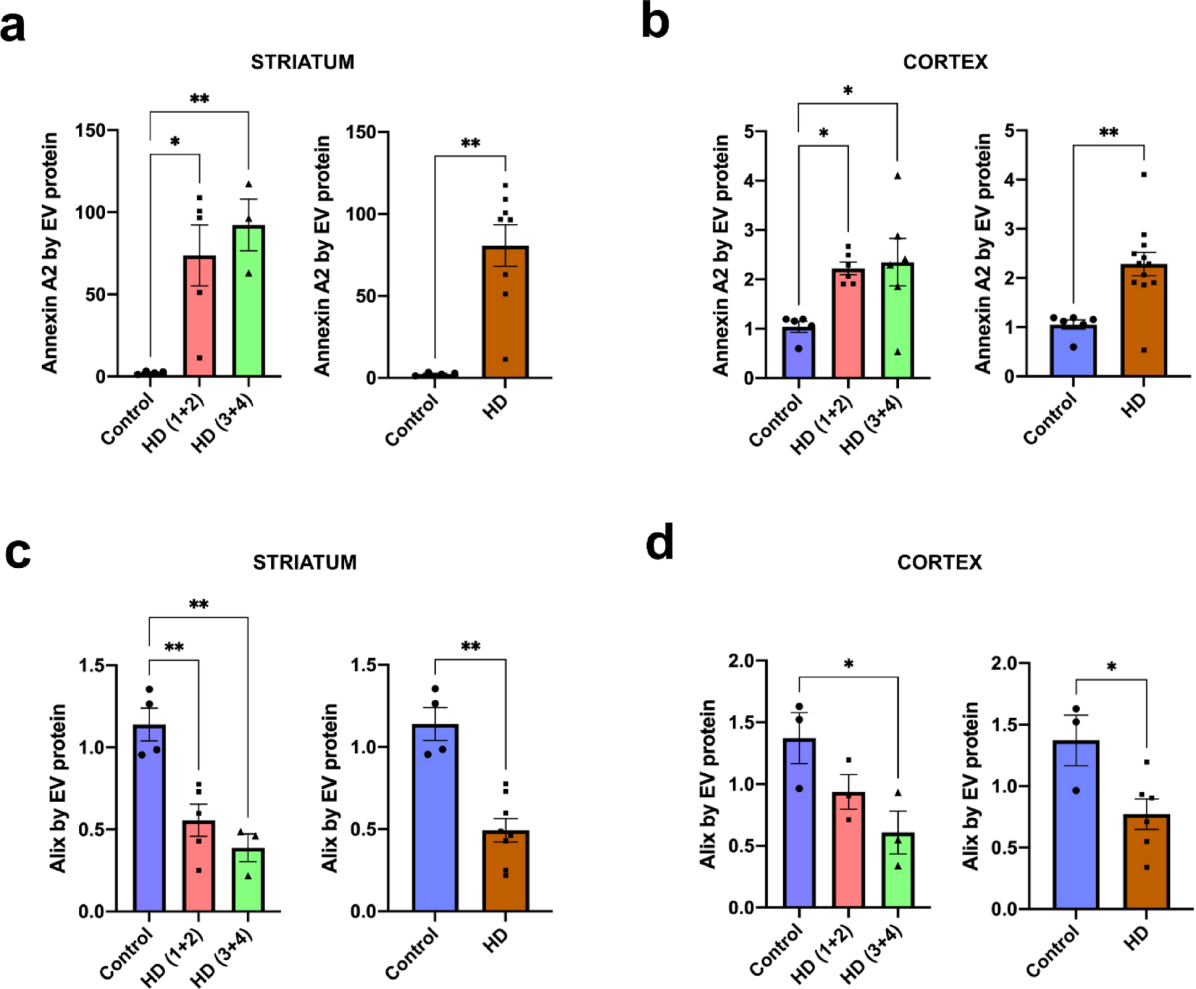
The content of Annexin A2 and Alix in HD brain EVs. Representation of the levels of Annexin A2 in striatum (**a**) and cortex (**b**) ectosomes normalized by EV protein. Significant main effects of the experimental group on Annexin A2 levels were detected in striatum and cortex ectosomes (*P* = 0.0069 and *P* = 0.0225, respectively). Pairwise comparison between HD and control showed significant differences in Annexin A2 expression in the striatum (*P* = 0.0040) and the cortex (*P* = 0.0032). Representation of the levels of Alix in the striatum (**c**) and cortex (**d**) exosomes normalized by EV protein. A significant main effect of the experimental group on Alix levels was detected in striatum exosomes and not in the cortex (*P* = 0.0014 and *P* = 0.0574, respectively). Pairwise comparison between HD and control showed significant differences in Alix expression in the striatum (*P* = 0.0040) and in the cortex (*P* = 0.0476). Ordinary one-way ANOVA followed by Tukey’s multiple comparisons test. When HD cases were combined Mann–Whitney tests were performed. In striatum: *n* = 4 Controls, *n* = 5 HD (1 + 2), *n* = 3 HD (3 + 4); and in cortex: *n* = 5 Controls, *n* = 6 HD(1 + 2), *n* = 6 HD(3 + 4) for ectosomes, and *n* = 3 per group for exosomes. Data is shown as mean ± SEM. *P < 0.05, **P < 0.01

To further explore the potential of Annexin A2 and Alix as subtype-specific markers of EVs in HD, we assessed the content of Annexin A2 and Alix per EV by normalizing the intensity of Western blot bands for each protein to the total amount of EV protein loaded. This allowed us to estimate the relative abundance of each protein per EV. We found that the level of Annexin A2 per EV in ectosomes was higher in the striatum (Fig. 5a) as well as in the cortex (Fig. 5b), in HD(1 + 2), HD(3 + 4) and in all HD cases combined, relative to controls. In the case of the levels of Alix in exosomes, HD cases contained less Alix than controls in the striatum (Fig. 5c), as well as in the cortex of HD(3 + 4) (Fig. 5d). Altogether, these results suggest subtype- and region-specific alterations in EV protein cargo in HD, reinforcing their potential value as biomarkers of disease-related changes.

### Reduced Alix levels in the brains of HD patients

**Fig. 6:**
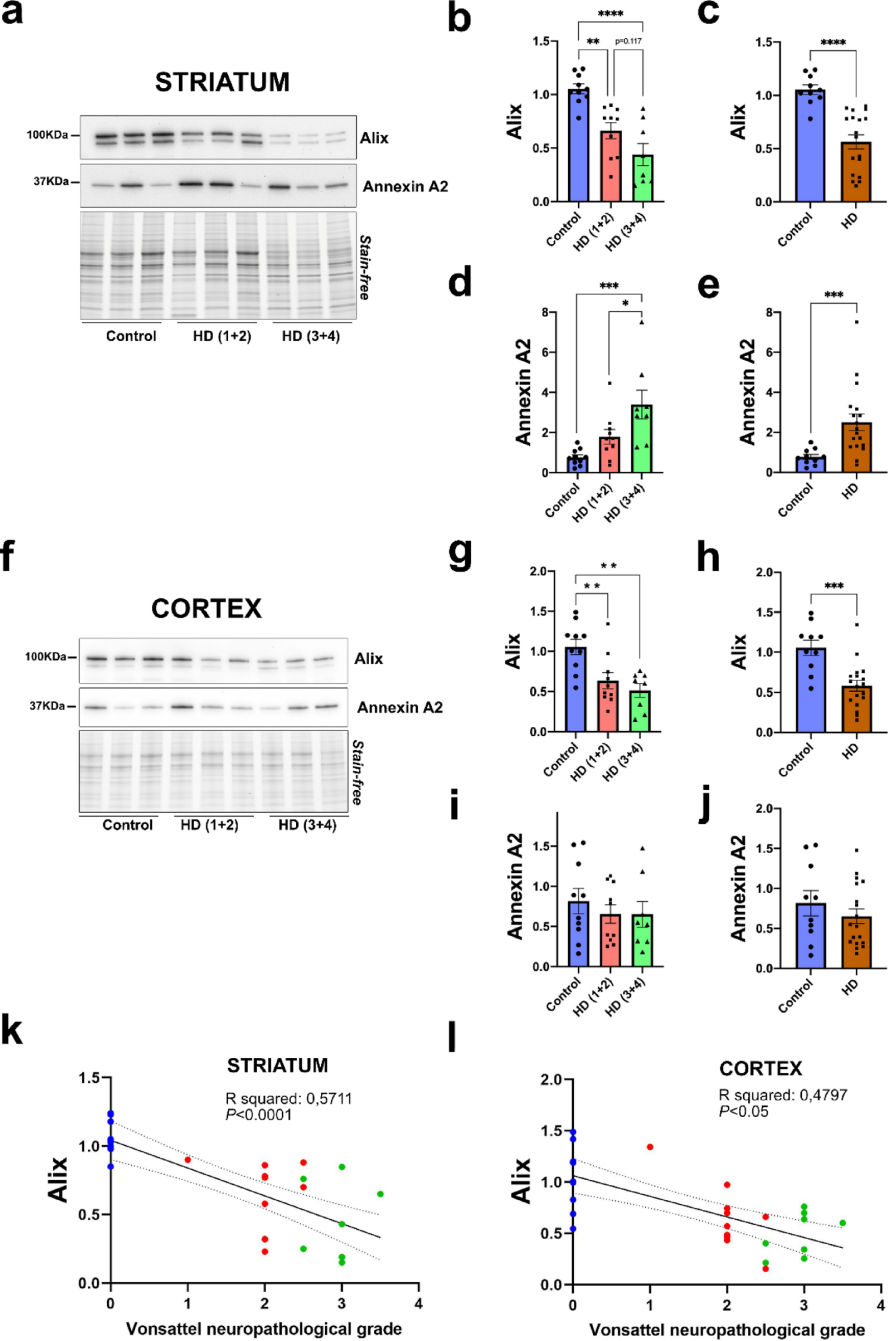
The levels of Alix and Annexin A2 in HD striatum and cortex. **a** Representative Western blot showing Alix, Annexin A2 in the striatum. Quantification of Alix (**b**) and Annexin A2 (**c**) in the three experimental groups. Significant main effects of the experimental group on Alix and Annexin A2 levels were detected in striatum (*P* < 0.0001 and *P* = 0.0010, respectively). Quantification of Alix (**d**) and Annexin A2 (**e**) in HD versus controls also show significant changes in the levels of Alix (*P* < 0.0001) and Annexin A2 (*P* = 0.0004). **f** Representative Western blot showing Alix and Annexin A2 in the cortex. Quantification of Alix (**g**) and Annexin A2 (**h**) in the three experimental groups. A significant main effect of the experimental group on Alix levels was detected in cortex (*P* = 0.0013) whereas no changes were detected on Annexin A2 levels (*P* = 0.648). Quantification of Alix **i** shows significant changes between HD and controls (*P* = 0.0007), unlike Annexin A2 (*P* = 0.4081) (**j**). The intensity of Alix or Annexin A2 bands was normalized to the total protein load estimated by the imaged stain-free gel intensity. The lower bands in the Vinculin image corresponds to Alix. Ordinary one-way ANOVA followed by Tukey’s multiple comparisons test. When HD cases were combined Mann–Whitney tests were performed. *n* = 10 Controls, *n* = 10 HD (1 + 2), *n* = 8 HD (3 + 4). Correlation between Alix level and brain Vonsattel neuropathological grade in striatum (**k**) and cortex (**l**). R-squared and *P*-values corrected by age are shown. Dashed-line indicates the 95% confidence bands of the best-fit line. Blue dots correspond to controls, red to HD(1 + 2) and green to HD(3 + 4)

Next, to better understand whether the altered EV cargo reflects a broader dysregulation of these proteins in HD brains we investigated if the altered loading of Annexin A2 and Alix in HD EVs was associated with changes in their expression levels in striatal and cortical homogenates. Our data indicated that the reduced loading of Alix in HD EVs was consistent with decreased Alix levels in these brain regions. Specifically, Alix levels were significantly reduced by 39% and 61% in the striatum of HD(1 + 2) and HD(3 + 4), respectively, compared to controls (Fig. 6a, b). When considering all HD cases combined, Alix levels were reduced 44% compared to controls (Fig. 6a, c). Regarding Annexin A2, we observed a significant increase in the striatum of HD(3 + 4) compared to both, controls and HD(1 + 2) (Fig. 6a, d), and also when considering all HD cases combined (Fig. 6a, e). Given that the levels of total protein in HD(3 + 4) brains were visually lower than the rest, additional analysis were performed by normalizing the data by Vinculin as loading control, instead of total protein. Whereas the results for Alix were unchanged (Supplementary Fig. 7a-c), Annexin A2 levels in HD brains were not significantly different from controls in this case, although the trend was kept (Supplementary Fig. 7a, d, e). In the cortex, Alix levels were significantly reduced by 43% and 55% in HD(1 + 2) and HD(3 + 4), respectively, compared to controls (Fig. 6f-h), whereas Annexin A2 levels remained unchanged (Fig. 6f, i, j). When Vinculin was used as the loading control, similar results were obtained (Supplementary Fig. 7f-j).

Given that Alix reductions were more pronounced in advanced HD brains, we assessed whether these alterations correlated with neuropathological severity. Notably, our data revealed a significant negative correlation between Alix levels and neuropathological severity, as determined by the Vonsattel grading system, in both the striatum (Fig. 6k) and the cortex (Fig. 6l) after correcting for age. Age corrections were applied given that Alix positively correlated with age in the cortex and showed a trend in the striatum homogenates (Supplementary Fig. 8).

### Reduced secretion of Alix-positive EVs by HD fibroblasts

**Fig. 7:**
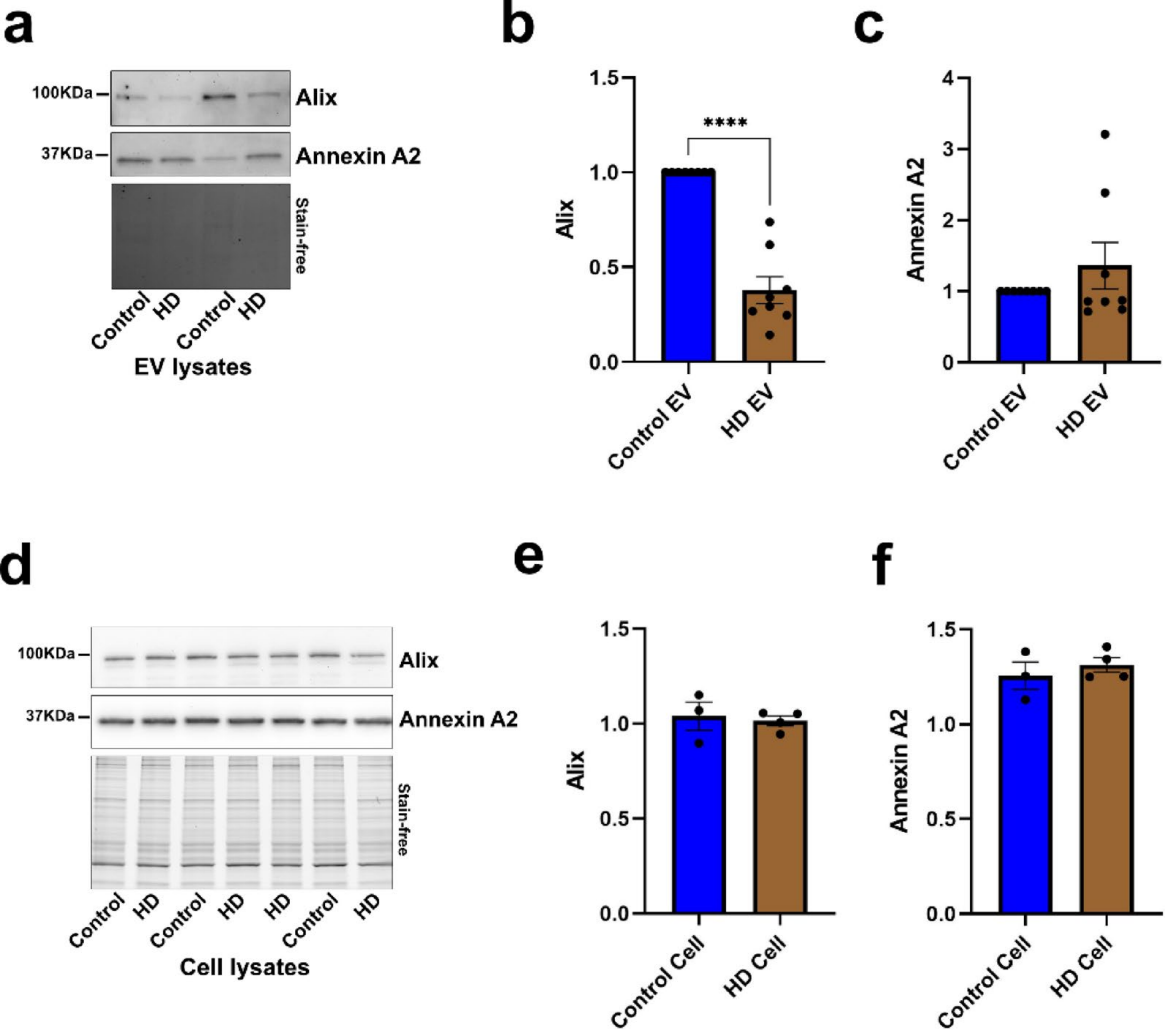
EV secretion in HD fibroblasts. Representative Western blot (**a**) and corresponding quantification of Alix (**b**) and Annexin A2 (**c**) in EVs isolated from the conditioned media of HD patients- and non-affected individuals-derived fibroblasts. The protein intensities were normalized by the total amount of protein measured in the fibroblast lysates. Unpaired t-test with Welch correction. *n* = 7 from 3 independent experiments. Representative Western blot (**d**) and corresponding quantification of Alix (**e**) and Annexin A2 (**f**) in fibroblast cell lysates from HD patients and non-mutation carriers. The intensity of Alix or Annexin A2 bands was normalized to the total protein load estimated by the imaged stain-free gel intensity. Unpaired t-test. Data is shown as mean ± SEM. ****P < 0.0001

Lastly, to further support our findings on altered EV cargo in HD, and given that endosomal abnormalities are also present in fibroblasts from HD mutation carriers [35], we performed in vitro studies to analyze EV secretion in the conditioned media from fibroblasts derived from three pre-symptomatic mutation-carriers, one symptomatic HD patient, and healthy controls. Our data indicate that HD fibroblasts secrete fewer Alix-positive EVs into the media compared to controls (Fig. 7a, b), whereas no significant changes were observed in Annexin A2 levels in EVs (Fig. 7a, c). Importantly, we observed that neither Alix (Fig. 7d, e), nor Annexin A2 (Fig. 7d, f) protein levels were altered in the fibroblast lysates compared to controls arguing that, unlike the brain, the changes seen at the EV secretion level are not directly driven by alterations in cellular protein levels.

## Discussion

Given the limited exosome research in HD compared to other neurodegenerative diseases, we aimed to investigate whether EV subtypes exhibit alterations in content and secretion by isolating EVs from the striatum and cortex of HTT-mutation carriers and controls. The purity of the EV preparations was confirmed by Western blotting, electron microscopy and NTA. These data were further supported by mass spectrometry analysis, which identified a high percentage of proteins previously detected in EVs. Furthermore, exosome proteomic analysis also revealed alterations in the level of key regulatory endosomal proteins in HD compared to controls. Further research is necessary to determine whether these altered proteins carried by EVs can reach the periphery offering opportunities for minimally invasive biomarker studies [36].

By exploring individually two distinct EV subpopulations in the brain, we have identified key alterations in the context of HD. Our data suggest that the level of ectosomes, characterized by the level of Annexin A2, increases early in HD cortex and reaches values closer to control levels as the neuropathology progresses. Furthermore, the levels of Annexin A2 per EV were also higher in HD EVs, a finding that was also supported by our mass spectrometry data. Given that EVs are potential vehicles for the removal of toxic proteins from cells, the increased level of these specific EVs may represent a cell response to early pathological changes that contribute to the elimination of potential pathological proteins such as tau or α-synuclein [37], proteins that were identified in our proteomic analysis. Even though mutant HTT has been previously found in association with EVs [38, 39], our mass spectrometry analysis only showed the presence of HTT in three control and two HD samples.

We have also shown that the EV population corresponding to denser EVs, exosomes, is reduced in HD striatum, regardless of the neuropathological stage. Additionally, Western blot analysis revealed a reduction in Alix levels per EV in HD exosomes compared to controls, a finding that was not detected in our proteomic analysis. This discrepancy may be due to the fact that mass spectrometry was performed on cortical EVs, whereas the most pronounced changes were observed in the striatum. The number of particles correlated with Alix levels in the striatum, suggesting that the reduced Alix signal likely reflects a lower number of secreted particles. However, because no significant correlation was observed in the cortex, we cannot exclude the possibility that a similar number of EVs are released but contain less Alix per vesicle.

Our findings of reduced exosome secretion in the striatum align with a recent report suggesting that crude EV levels are decreased in HD versus control conditions in mouse striatal cells, which was also associated to changes in the physical features of exosomes [40]. Furthermore, less exosome secretion in the striatum of the HD140Q Knock-in mouse model of the disease has been reported [41]. To our knowledge, this is the first study to describe such alterations in human brains, providing translational support for previous observations in animal models. Endosomal impairment has been associated with alterations in the exosomal pathway, specifically an increase in exosome secretion in Down syndrome patients [11]. Notably, reducing exosome generation by knocking down CD63 worsened endosomal pathology in cultured Down syndrome fibroblasts [11], suggesting that flux through the exosomal pathway is a crucial regulator of endosomal compartments. Therefore, reduced exosome release in the HD brain may contribute to the endosomal-lysosomal pathway alterations observed in the disease [6, 7, 35, 42], similar to what was previously observed in carriers of Apolipoprotein E4 [43], the most prominent risk gene for Alzheimer’s disease (AD). In addition, given our data of lower Alix protein levels per EV in HD compared to controls, and the well-established role of Alix in exosome biogenesis [44], it is likely that the altered formation of intraluminal vesicles at the MVB leads to the release of less exosomes into the extracellular space. However, it is also possible that less MVB content exocytosis occurs in favor of fusion with lysosomes, as has been recently demonstrated [10].

Our results also indicate that, beyond EVs, Alix protein levels are reduced in HD striatum and cortex homogenates compared to controls, and that Alix levels correlate with neuropathological severity, as assessed by the Vonsattel grading system. Based on these findings, Alix may serve as a novel neuropathological marker in HD.

In addition to the alterations observed in the HD brain, Alix may also represent a promising candidate for biomarker development in biofluids. Although our present study focused on brain-derived samples, the assessment of Alix levels in plasma- or serum-derived EVs could provide valuable insights into disease-related changes accessible through minimally invasive sampling. Recent work by Bras et al. [45] examined exosomes and ectosomes isolated from plasma of HD patients and controls. In agreement with our data, they report higher Alix enrichment in exosomes than in ectosomes. However, they did not observe differences in Alix levels between disease and control groups. Similarly, Herrero-Lorenzo et al. [46] presented data from plasma EVs at different disease stages without apparent alterations in Alix levels, though their study did not specifically quantify Alix. The lack of significant findings in these preliminary studies may reflect small cohort sizes and methodological variability rather than the absence of biological relevance. Given the complexity of plasma composition and the potential contribution of both EV-associated and soluble Alix, larger and more targeted studies will be required to determine its prognostic potential. Furthermore, given the ability of EVs to cross the blood–brain barrier [47], Alix could potentially function as a peripheral reporter of brain pathology as a canonical component of EVs. Indeed, significant efforts are currently focused on identifying markers for immunocapturing brain-derived EVs from blood [36, 48, 49]. Additionally, Alix can be present in soluble form in blood and other biofluids, such as cerebrospinal fluid, potentially serving as a biomarker by itself, without being associated with EVs. Notably, Alix levels measured directly in serum were found to be reduced in AD patients and correlated with cognitive impairment [50], which opens the possibility of Alix as a potential soluble biomarker also in HD.

Beyond its role in exosome biogenesis, some studies have shown that Alix knockout mice exhibit reduced brain volume, suggesting that Alix is required for normal brain development [51, 52]. Thus, alterations in Alix may contribute to the impaired neurodevelopment observed in HD [53]. It remains to be determined whether Alix levels are also reduced in the brains of younger, non-symptomatic HTT mutation carriers. In addition, Alix is involved in mediating apoptosis neuronal death programs, likely mediated via signaling through the endosomal pathway [54–56], and therefore may have also an impact in HD pathogenesis.

Even though the data presented here corresponds to two key brain areas from the same individuals, one of the main limitations of this study is that, given the high amount of tissue needed for EV isolation, the number of brain samples tested for the EV work is relatively small. In addition, because we analyzed bulk brain EVs, information on the cell origin of the changes is lacking. It is likely that specific brain cells contribute more than others to the observed changes and this would be worth of further research. Of note, the neuropathological examination of some post-mortem tissues used in this study revealed the presence of tau pathology categorized with the Braak tau staging. Thus, even though we did not observe significant differences within the HD brains displaying different Braak stages, we cannot rule out the possibility that tau levels may have an impact on EVs in HD brains.

Complementing our results in the HD brain, we have detected reduced levels of Alix in EVs secreted by HD fibroblasts in vitro. Importantly, three out of four fibroblast cells that we used, corresponded to premanifest mutation carriers, arguing for changes that occur early in the disease. Unlike in the brain, we did not find alterations in total Alix or Annexin A2 levels in HD fibroblast lysates, suggesting that such relationships may be brain region–specific and reflect neuronal vulnerability or local disease burden. A limitation of this study is that endo-lysosomal alterations were not directly characterized in our fibroblast cultures. Although prior studies have demonstrated such defects in HD fibroblasts, cellular heterogeneity among primary human samples may influence the phenotype. Consequently, our conclusions regarding the relationship between Alix levels and endo-lysosomal dysfunction should be considered preliminary.

A recent study has also explored the EV secretion in HD fibroblasts and controls and reported enhanced secretion in fibroblasts from manifest HD patients compared to controls [57]. Although the authors in this study did not assess the specific contributions of EV subpopulations, our findings suggest that the increased EV secretion may originate from a population distinct from exosomes, such as other small EVs or mitovesicles [21]. Finally, although skin fibroblasts as the chosen cell model might not fully represent what happens in the brain, our study reveals similarities with the human brain in terms of Alix-positive EV secretion. Furthermore, a recent study compared EVs from fibroblasts and neural stem cells from the same patients and found that the data between the two systems were in agreement [57]. In any case, complementary in vitro studies using stem cells differentiated into specific brain cells are encouraged in order to elucidate the mechanisms behind the EV alterations that we have seen in the HD brains.

## Conclusions

Our findings provide new insights into the pathophysiology of EVs in HD brains, with alterations in key proteins, Alix and Annexin A2, which highlights brain EVs as potential targets to restore cellular homeostasis. Additionally, we have identified Alix as a novel marker of neuropathology in HD, suggesting that Alix, either as a cytosolic protein or as a key component of EVs, could serve as a valuable biomarker for disease progression. Thus, these discoveries open new avenues for the exploration of Alix in both prognostic and therapeutic contexts.

## Supplementary Information

Supplementary Material 1

Supplementary Material 2: Table S1. List of proteins identified by mass spectrometry analysis that met the significance threshold of *p* < 0.01 with a fold change >2.8. It includes protein identifiers and the category where the differences were found: HD1vsC: HD(1+2) versus Control; HD3vsC: HD(3+4) versus Control; or HD3vHD1: HD(3+4) versus HD(1+2).

## Data Availability

The raw proteomics data have been deposited to the PRIDE repository with the project accession number PXD060574.

## Abbreviations

AD: Alzheimer’s disease
EM: Electron microscopy
ESCRT: Endosomal sorting complex required for transport
EVs: Extracellular vesicles
Fr1-3: EV iodixanol fractions 1 to 3
Fr4-7: EV iodixanol fractions 4 to 7
HD: Huntington’s disease
HD(1 + 2): HD brains with Vonsattel grades 1 or 2
HD(3 + 4): HD brains with Vonsattel grades 3 or 4
HTT: Huntingtin
MVB: Multivesicular body
NTA: Nanoparticle tracking analysis
SEM: Standard error mean
