# Supplementary material for: The profiling of extracellular vesicle subtypes in Huntington’s disease brains identifies Alix as a novel marker of neuropathology
Source: Acta Neuropathol Commun. 2025 Dec 8;14:39. doi: 10.1186/s40478-025-02187-6 (PMC12896033; doi:10.1186/s40478-025-02187-6)
Supplement: Supplementary file 1 — Supplementary Material 1 [file 40478_2025_2187_MOESM1_ESM.docx]

**Supplementary figures**

**
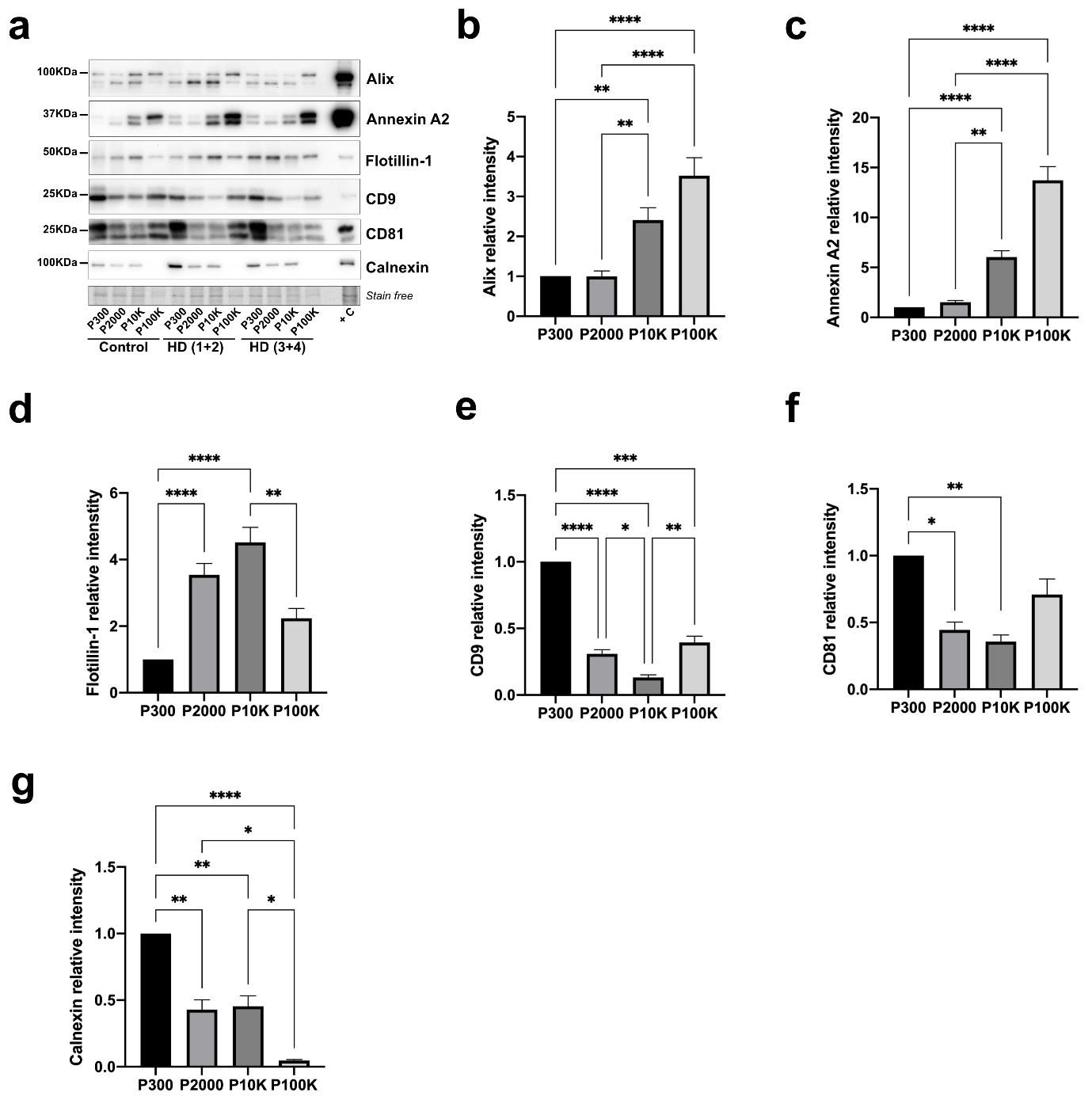
**

**Supplementary Fig. 1. Enrichment of EV proteins in the crude EVs.** (**a**) Representative Western blot and corresponding quantification of the EV markers Alix (**b**), Annexin A2 (**c**), Flotillin-1 (**d**) CD9 (**e**) and CD81 (f), and the intracellular marker Calnexin (**g**). Only the upper band, corresponding to the predicted molecular weight of Alix (96 KDa) was quantified. +C = cell lysate used as positive control. *n*=18 (except for CD81, for which *n*= 6 ) corresponding to the cortex samples used for EV isolation and mass spectrometry. Kruskal-Wallis test with Dunn’s multiple comparisons. Data is shown as mean ± SEM. * *P* < 0.05, ** *P* < 0.01, *** *P* < 0.001, **** *P* < 0.0001

**
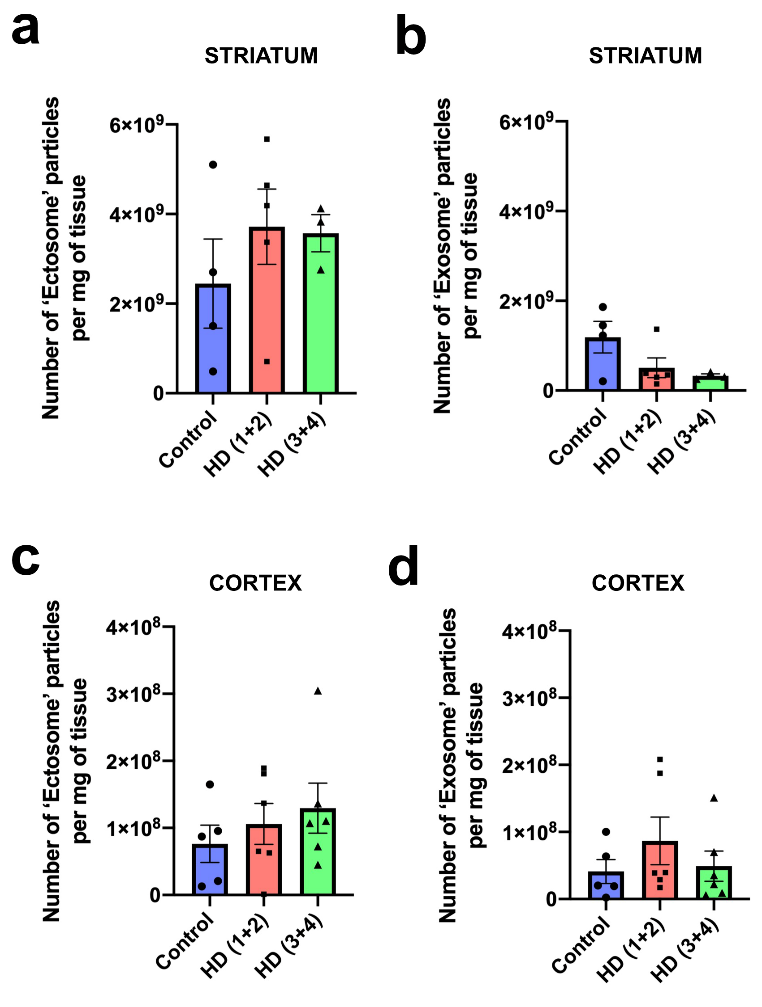
**

**Supplementary Fig. 2. Total number of particles in EV subpopulations from HD brains.** Number of particles for **‘**Ectosomes’ (Fr1-3 EVs) and ‘Exosomes’ (Fr-4-7 EVs**)** of the three experimental groups were estimated by NTA in the striatum (**a** and **b**, respectively) and cortex (**c** and **d**, respectively). Ordinary one-way ANOVA followed by Tukey’s multiple comparisons test. For striatum analysis: *n*=4 Controls, *n* =5 HD (1+2), *n* =3 HD (3+4); for cortex analysis *n*=5 Controls, *n* =6 HD (1+2), *n* =6 HD (3+4). Data is shown as mean ± SEM.


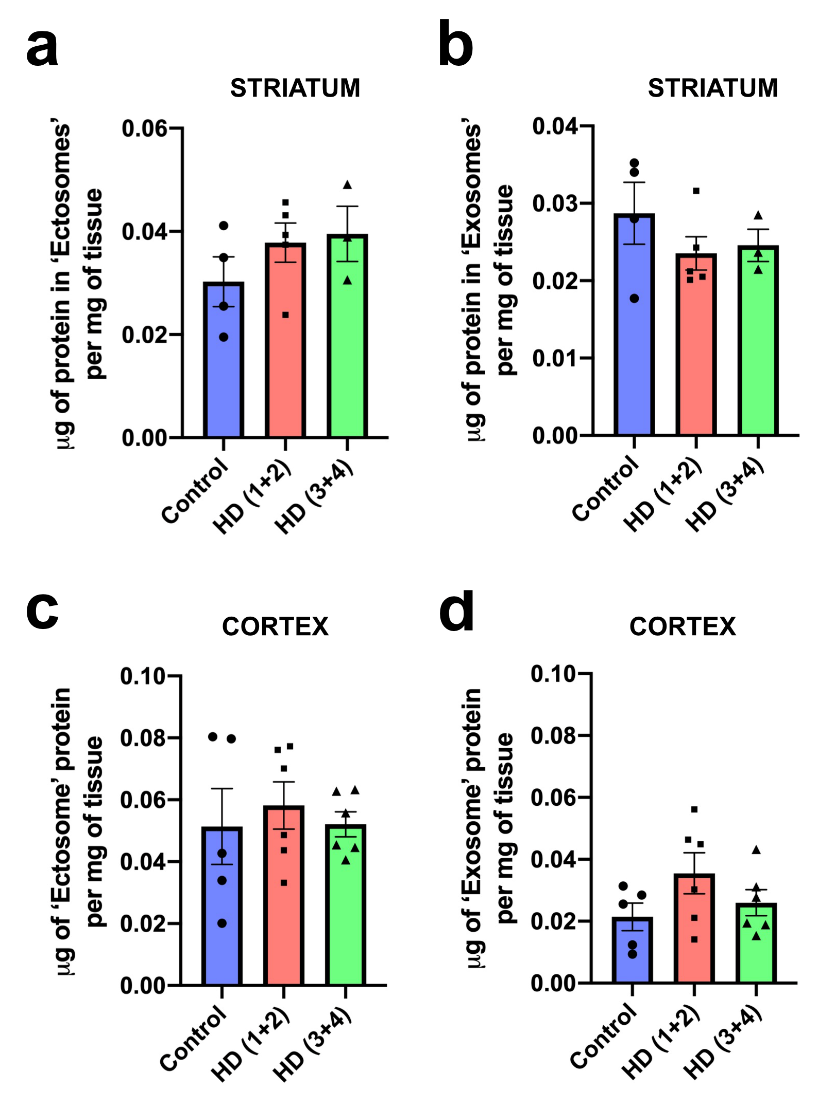


**Supplementary Fig. 3. Total protein estimation in EV subpopulations from HD brains.** Protein concentration values for ‘Ectosomes’ (Fr1-3 EVs) and ‘Exosomes’ (Fr-4-7 EVs**)** of the three experimental groups were estimated by BCA in the striatum (**a** and **b**, respectively) and cortex (**c** and **d**, respectively). Ordinary one-way ANOVA followed by Tukey’s multiple comparisons test. For striatum analysis: *n*=4 Controls, *n* =5 HD (1+2), *n* =3 HD (3+4); for cortex analysis *n*=5 Controls, *n* =6 HD (1+2), *n* =6 HD (3+4). Data is shown as mean ± SEM.

**
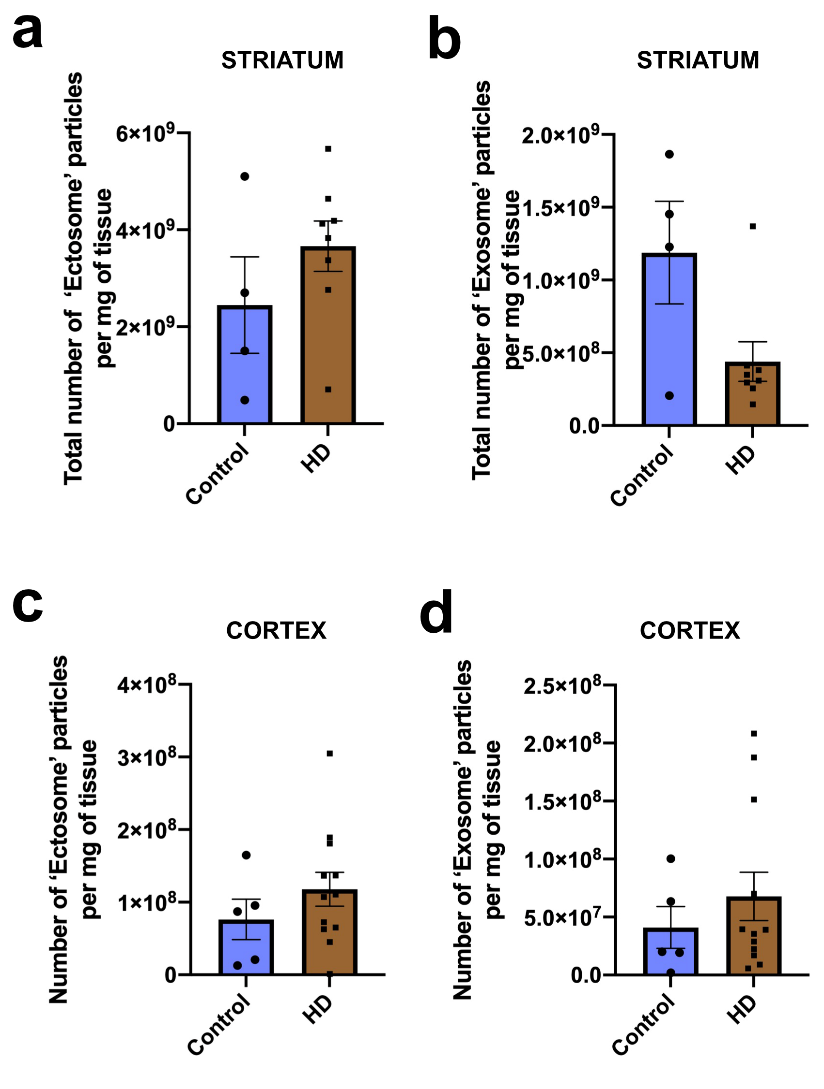
**

**Supplementary Fig. 4. Total number of particles in EV subpopulations from HD brains.** Number of particles for **‘**Ectosomes’ (Fr1-3 EVs) and ‘Exosomes’ (Fr-4-7 EVs**)** of HD and controls estimated by NTA in the striatum (**a** and **b**, respectively) and cortex (**c** and **d**, respectively). Mann-Whitney tests were performed. For striatum analysis: *n*=4 Controls, *n* =8 HD; for cortex analysis *n*=5 Controls, *n* =12 HD. Data is shown as mean ± SEM.


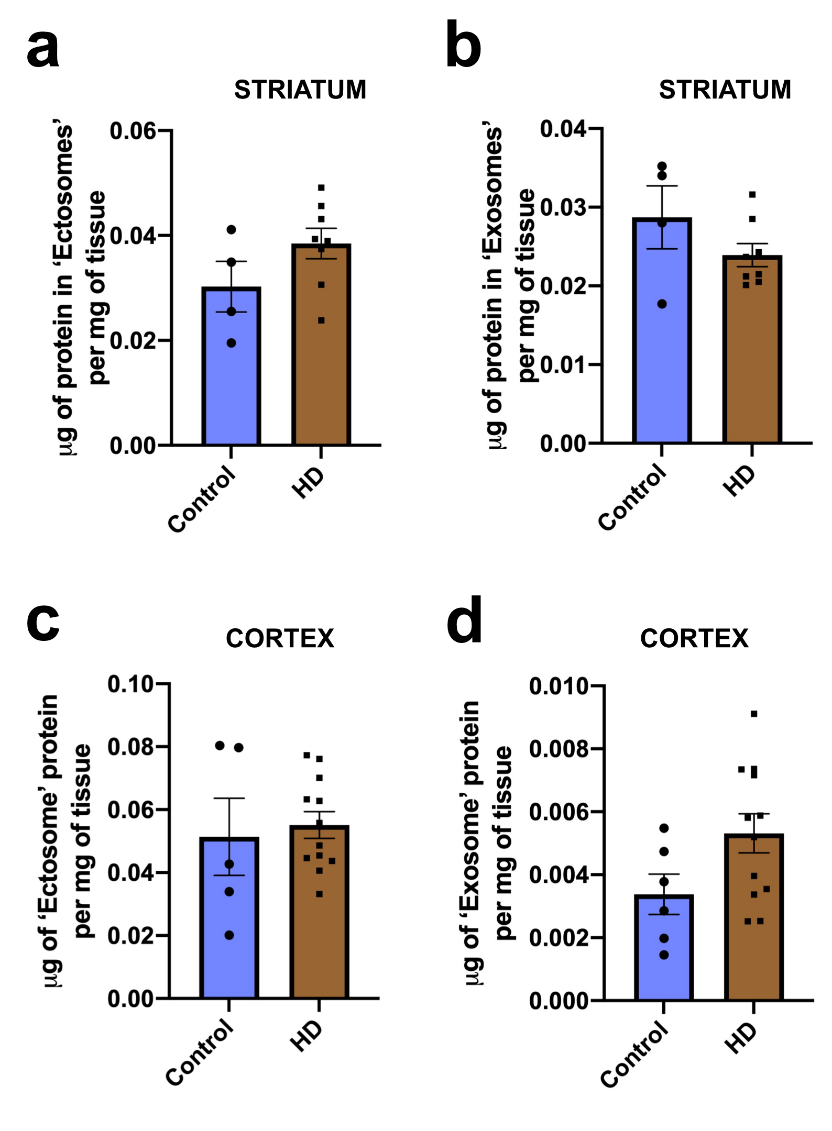


**Supplementary Fig. 5. Total protein estimation in EV subpopulations from HD brains.** Protein concentration values for ‘Ectosomes’ (Fr1-3 EVs) and ‘Exosomes’ (Fr-4-7 EVs**)** of HD vs. controls were estimated by BCA in the striatum (**a** and **b**, respectively) and cortex (**c** and **d**, respectively). Mann-Whitney tests were performed. For striatum analysis: *n*=4 Controls, *n*=8 HD; for cortex analysis *n*=5 Controls, *n* =12 HD. Data is shown as mean ± SEM.


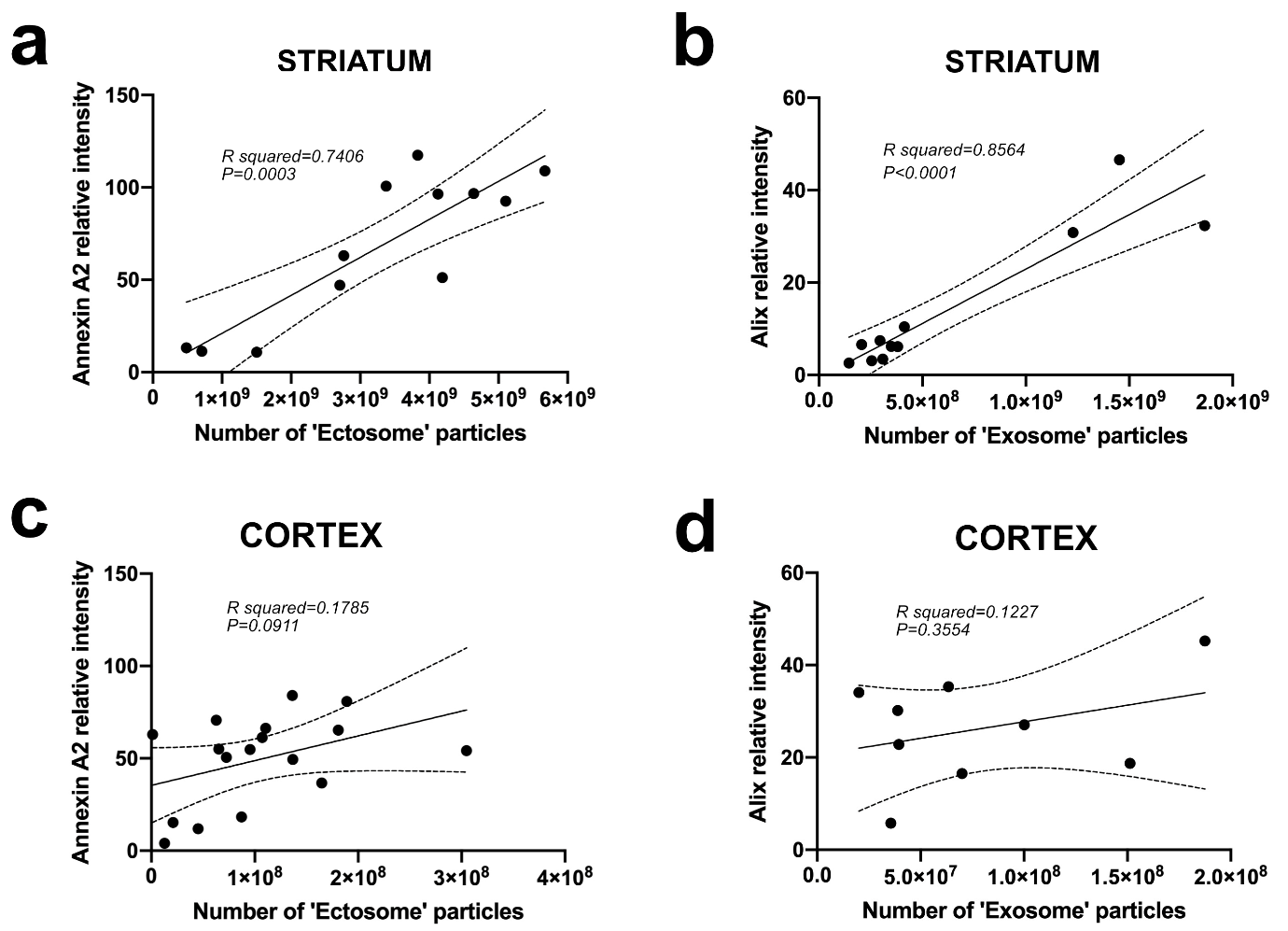


**Supplementary Fig. 6. Correlations between the number of particles and the level of Annexin A2 or Alix.** Correlations between Annexin A2 and the number of ‘Ectosome’ particles (**a**) and between Alix and the number of ‘Exosome’ particles (**b**) in the striatum. Correlations between Annexin A2 and the number of ‘Ectosome’ particles (**c**) and between Alix and the number of ‘Exosome’ particles in the cortex (**d**). R-squared and *P*-values are shown. Dashed-line indicates the 95% confidence bands of the best-fit line. *n*= 12 (Annexin A2 in striatum), *n*=11 (Alix in striatum), *n*=17 (Annexin A2 in cortex), and *n*= 9 (Alix in cortex).


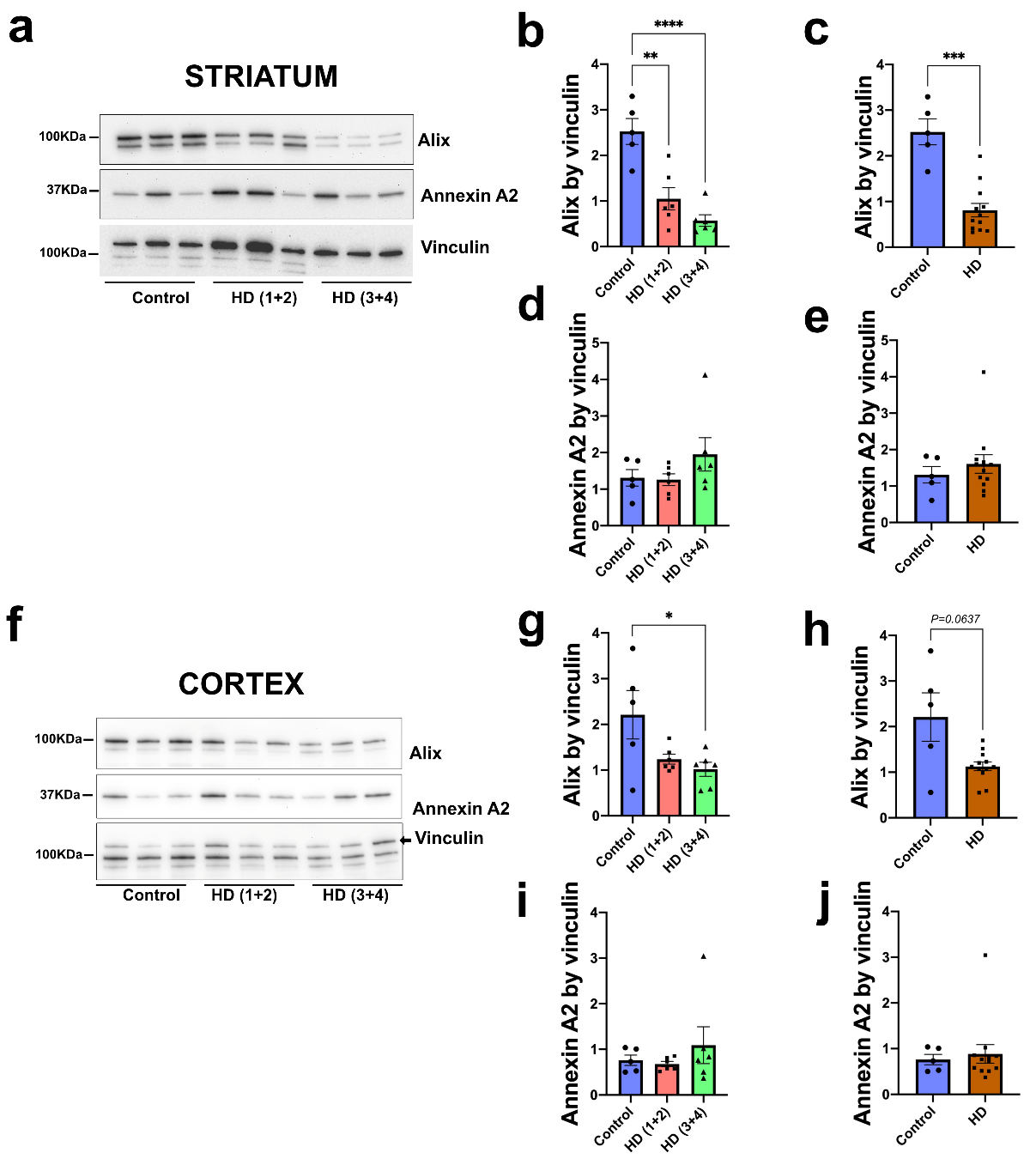


**Supplementary Fig. 7. Alix and Annexin A2 levels in HD striatum and cortex homogenates normalized by Vinculin. (a)** Representative Western blot showing Alix, Annexin A2 and Vinculin in the striatum and corresponding quantification of Alix **(b,c)** and Annexin A2 **(d,e)** normalized by Vinculin. **(a)** Representative Western blot showing Alix, Annexin A2 and Vinculin in the cortex and corresponding quantification of Alix **(g,h)** and Annexin A2 **(i,j)** normalized by Vinculin. Ordinary one-way ANOVA followed by Tukey’s multiple comparisons test was performed when three experimental groups were compared. When HD cases were combined Mann-Whitney tests were performed. *n* =5 Controls, *n* =6 HD (1+2), *n* =6 HD (3+4).

**
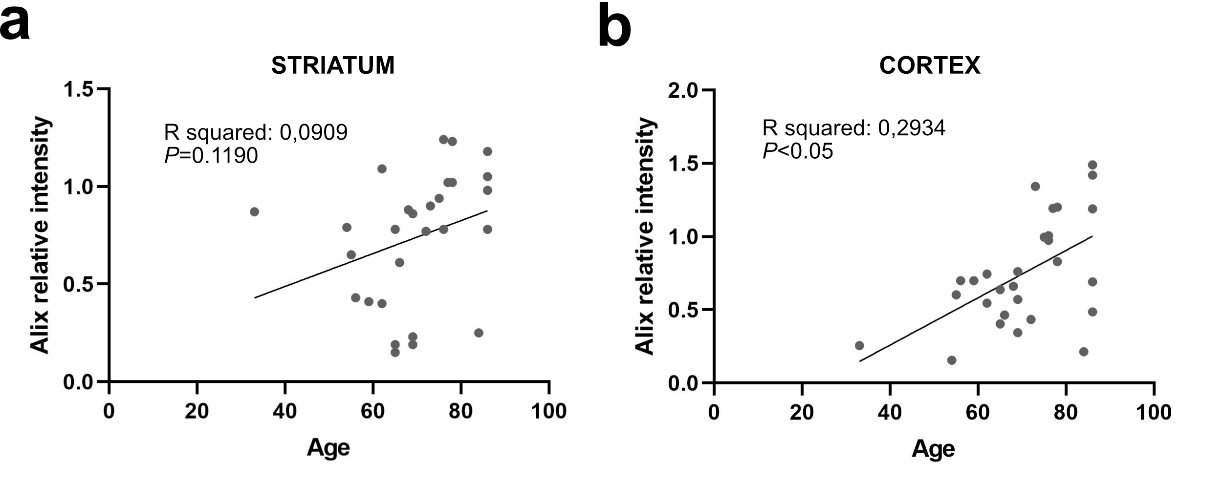
**

**Supplementary Fig. 8. Alix correlations with age.** Correlation between Alix level and age in striatum **(a)** and cortex **(b)**. R-squared and *P*-values are shown. *n* =10 Controls, *n* =10 HD (1+2), *n* =8 HD (3+4).
